# Bacterial diversity and chemical ecology of natural product–producing bacteria from Great Salt Lake sediment

**DOI:** 10.1093/ismeco/ycae029

**Published:** 2024-03-04

**Authors:** Elijah R Bring Horvath, William J Brazelton, Min Cheol Kim, Reiko Cullum, Matthew A Mulvey, William Fenical, Jaclyn M Winter

**Affiliations:** Department of Pharmacology and Toxicology, University of Utah, Salt Lake City, UT 84112, United States; School of Biological Sciences, University of Utah, Salt Lake City, UT 84112, United States; Department of Medicinal Chemistry, University of Utah, Salt Lake City, UT 84112, United States; School of Biological Sciences, University of Utah, Salt Lake City, UT 84112, United States; Center for Marine Biotechnology and Biomedicine, Scripps Institution of Oceanography, University of California at San Diego, CA 92093, United States; Center for Marine Biotechnology and Biomedicine, Scripps Institution of Oceanography, University of California at San Diego, CA 92093, United States; School of Biological Sciences, University of Utah, Salt Lake City, UT 84112, United States; Henry Eyring Center for Cell and Genome Science, University of Utah, Salt Lake City, UT 84112, United States; Center for Marine Biotechnology and Biomedicine, Scripps Institution of Oceanography, University of California at San Diego, CA 92093, United States; Skaggs School of Pharmacy and Pharmaceutical Sciences, University of California at San Diego, CA 92093, United States; Moores Comprehensive Cancer Center, University of California at San Diego, CA 92037, United States; Department of Pharmacology and Toxicology, University of Utah, Salt Lake City, UT 84112, United States

**Keywords:** Great Salt Lake, halophiles, natural products, Actinomycetota, chemical ecology

## Abstract

Great Salt Lake (GSL), located northwest of Salt Lake City, UT, is the largest terminal lake in the USA. While the average salinity of seawater is ~3.3%, the salinity in GSL ranges between 5% and 28%. In addition to being a hypersaline environment, GSL also contains toxic concentrations of heavy metals, such as arsenic, mercury, and lead. The extreme environment of GSL makes it an intriguing subject of study, both for its unique microbiome and its potential to harbor novel natural product–producing bacteria, which could be used as resources for the discovery of biologically active compounds. Though work has been done to survey and catalog bacteria found in GSL, the Lake’s microbiome is largely unexplored, and little to no work has been done to characterize the natural product potential of GSL microbes. Here, we investigate the bacterial diversity of two important regions within GSL, describe the first genomic characterization of Actinomycetota isolated from GSL sediment, including the identification of two new Actinomycetota species, and provide the first survey of the natural product potential of GSL bacteria.

## Introduction

Great Salt Lake (GSL), located in the state of UT, is the largest saltwater body in the USA and represents one of the most hypersaline and extreme environments in the world [1, 2]. Its area spans five counties, including Weber, Box Elder, Salt Lake, Tooele, and Davis, and covers ~4400 km^2^, though its area is rapidly shrinking due to climate change and water diversion [1, 3, 4]. Due to its location in the Great Basin of the Intermountain West, GSL experiences temperature fluctuations ranging from −5°C in the winter months to >35°C in the summer months. As GSL is a terminal lake, meaning that water flowing into it only leaves by evaporation, and the minerals, ions, and salts that enter the lake are retained and concentrated. A railroad causeway constructed in the 1950s physically divides GSL into two main regions: the North and South Arms. During the snowpack melt in the spring, most of the fresh water is brought into the South Arm and because of this, the salinity can range from 5% to >15% in the South Arm and often reaches ≥27% in the North Arm, which receives little to no fresh water inflow [5].

The chemical makeup of GSL closely resembles that of typical ocean water, with sodium and chloride being the primary ions, followed by sulfate, magnesium, calcium, and potassium [1]. Although the North Arm exhibits higher ion abundance than the South Arm, their ion compositions are similar [6, 7]. However, the considerable differences in salinity significantly influence the microbiota between the two arms, creating two sub ecosystems within the lake [1, 2, 7, 8]. A broader microbial diversity is observed in the South Arm compared to the North Arm, and this is primarily attributed to the differences in salinity [9, 10]. Interestingly, early studies found that the majority of halophiles isolated from GSL habitats were obligate halophiles rather than halotolerant transplants [11]. Media studies using nutrient agar with varying concentrations of GSL water, Pacific Ocean water, or distilled water revealed a notable decrease in colony-forming units as the percentage of GSL water was reduced in the media [12]. These findings suggest that despite similar ion concentrations between GSL and typical ocean water, the microbiology between these two environments is quite different.

Aside from being a hypersaline environment, GSL also exhibits elevated levels of heavy metals and metalloids, including mercury, arsenic, cadmium, and lead [13]. Due to the extreme environments of GSL, microorganisms surviving and thriving in the North and South Arms have been described as polyextremophiles, as they have adapted to not only toxic levels of heavy metals and extreme osmotic stress but also to extreme seasonal temperature fluctuations [1, 9]. To thrive in these harsh environments, GSL microorganisms may evolve and employ different methods for survival, such as heavy metal efflux, degradation of toxic compounds [14], and production of specialized small molecules. Of particular interest to us is the natural product potential of GSL bacteria.

Natural products are small molecules produced in nature and represent some of the most important pharmaceutical agents used in human health care [15–17]. This especially holds true with anti-infective agents, as many of the current clinically significant antibiotics are natural products or derivatives thereof. However, antibiotic resistance is on the rise and it has been recognized by the World Health Organization as a leading global health issue [18, 19], which needs to be urgently addressed. Antibiotic discovery over the last few decades has relied heavily on chemically modifying the scaffolds of known antibiotic agents. Therefore, the identification of bioactive natural products possessing new chemical scaffolds is a promising approach for the discovery of antibiotics with novel modes of action. Unique environments can influence the chemical diversity of natural products, and microorganisms isolated from extreme environments serve as ideal resources for drug discovery efforts. Furthermore, because GSL is a terminal lake and serves as the endpoint of wastewater treatment runoff from a major metropolitan center, we expect pathogenic microbes residing there to be a reservoir of antibiotic resistance genes. Therefore, native GSL microbes may be producing novel antibiotic agents to compete with these pathogens, which could make them an ideal resource for the identification of new natural products with novel scaffolds and new mechanisms of action.

To prioritize strains for downstream fermentation studies and streamline the labor-intensive natural product discovery pipeline when working with large numbers of bacterial isolates, robust bioinformatic and genomic tools can complement the bioactivity-guided isolation efforts [20–22]. In the producing organism, the genes encoding the enzymatic machinery used to assemble small molecules are typically clustered together within a chromosome or on extra-chromosomal genetic elements. By correlating genetic information to protein function, chemical logic can be used to connect a natural product to its respective biosynthetic gene cluster (BGC) [23]. However, little information on bacterial populations and even less on bacterial genomes has been reported from GSL [1, 8, 9]. Between the end of the 19th century and early 20th century, culture-dependent approaches were used to identify bacteria within GSL, with most research focusing on planktonic communities from the water column [11, 24–29] rather than sediment-derived microbial populations [10]. However, after construction of the causeway in the 1950s, culture-independent approaches became the predominant method for monitoring the composition of planktonic communities in the water column, as perturbations within the community can negatively affect the brine shrimp industry [8, 10, 27, 29, 30]. While the culture-dependent and culture-independent approaches were used to study and understand the microbial ecology of GSL, no genomic studies have been published to date and little is known about the natural product potential of the microorganisms residing in the hypersaline environment. Thus, it is imperative that we further characterize this unique and understudied ecosystem, with a focus on expanding our knowledge of what sediment-dwelling microbes are present using both culture-dependent and independent methods. Just as importantly, we must also assess GSL bacteria for their potential to produce novel natural products. Due to severe drought-related shrinkage of the Lake, it is imperative that we perform these studies before this invaluable resource is gone [1, 3]. Here, we investigate the microbial diversity in the South Arm of GSL as well as characterize the natural product potential of Actinomycetota strains isolated from the GSL sediment.

## Materials and methods

### Collection and isolation of bacteria strains

Sediment samples were collected in sterile Whirl-Pak bags or sterile Falcon tubes from the South Arm of GSL at four Black Rock Beach (BRB) and four Marina sites during the summer of 2017. For each collection site, samples were taken ca. 2.5 m apart in a linear sampling pattern. *Saccharomonospora* sp. GSL17-019 and *Streptomyces* sp. GSL17-113 were isolated from the Marina sediment that was desiccated for 72 h in a biological safety cabinet. Desiccated sediment, ~0.5 g, was added to yeast–peptone–mannitol (YPM) agar plates (per liter: 4 g mannitol, 2 g yeast extract, 2 g peptone, 18 g agar, and 42 g Instant Ocean Aquarium Sea Salt Mixture, Spectrum Brands, USA). Plates were incubated at 30°C for up to 90 days, and bacterial colonies were subcultured on YPM media until pure isolates were obtained. The 16S rRNA gene sequencing was used to identify *Saccharomonospora* sp. GSL17-019, *Streptomyces* sp. GSL17-113, and *Streptomyces* sp. GSL17-111 using NCBI Blast/Blast+ [31] via multiBLAST (https://github.com/ERBringHorvath/multiBLAST) (GenBank accession for the *Escherichia coli* reference 16S rRNA sequence is CP082835.1).

### Environmental sequencing of 16S ribosomal ribonucleic acid genes

DNA preparations of GSL sediment were carried out using a FastDNA Spin Kit (MP Biomedicals, Cat. No.: 116540-600). Sequencing of amplicons generated from 16S rRNA genes was performed at the Genomics Core Facility at Michigan State University on an Illumina MiSeq instrument using dual-indexed Illumina fusion primers targeting the V4 region of the 16S rRNA gene [32]. Amplicon sequence variants (ASVs) were inferred from 16S rRNA amplicon sequences with DADA2 v. 1.10.1 [33] after the removal of primer sequences with cutadapt v. 1.15 [34]. Taxonomic classification of ASVs was performed with DADA2 using the SILVA reference alignment (SSURefv132) and taxonomy outline [35, 36]. The ordination plot was generated with PhyloSeq v. 1.26.1 [22] using principal coordinate analysis (PCoA) ordination of Bray–Curtis dissimilarity values.

### Statistical analysis and data visualization

Multivariate plot and DESeq2 analyses were carried out using PhyloSeq v.1.42.0 [21, 22] in RStudio [37] using R v.4.3.1. DESeq2 v.1.38.3 was used to compare ASV data from the four BRB and four Marina sampling sites and graph differential abundance. During the DESeq2 analysis, each of the four collection sites from both BRB and the Marina were treated as replicates of the primary collection region to obtain a BRB versus Marina comparison. Significance was determined using a Wald test with a Benjamini and Hochberg adjusted *P*-value. Only ASVs meeting the minimal cut-off of an adjusted *P*-value of *P* > .01 were included in the results. Bar plots were created in RStudio using ggplot2 v.3.4.2 and ggbreak v.0.1.1 [38]. PhyloSeq-generated taxonomy classifications were updated to reflect current literature using the NCBI taxonomy browser. Phylogenetic trees were constructed with autoMLST using IQ-TREE Ultrafast Bootstrap analysis of 1000 replicates. The autoMLST-generated phylogenetic tree was exported as a Newick file and was used to generate final figures in R using ggtree v.3.8.0 [39].

### Genome sequencing and genome mining of Great Salt Lake genomes

For *Saccharomonospora* sp. GSL17-019 and *Streptomyces* sp. GSL17-113, construction of genomic libraries and sequencing were performed at the High-Throughput Genomics Center in the Huntsman Cancer Institute at the University of Utah. A PCR-free NEBNext Ultra II DNA library was generated and sequenced using NovaSeq S4 Reagent Kit v1.5 (150 × 150 bp and 2500 M read-pairs/lane). Adapter sequences and PhiX were removed from all reads with BBDuk, part of the BBtools suite, V35.85 [40]. Quality trimming was performed with seq-qc as previously described [41], and sequences were assembled using SPAdes [42]. Contigs shorter than 200 bp were trimmed using a custom Python script. CheckM [43] was used to assess assembly completeness. The GSL17-113 assembly consists of 1051 contigs with an average coverage depth of 1219×, an N50 value of 314 799, L50 value of 7, 99.64% completeness, and a contamination score of 0.53. The GSL17-019 assembly consists of 6254 contigs with an average coverage depth of 1331×, an N50 value of 363 195, L50 value of 4, 100% genome completeness, and a contamination score of 0. For *Streptomyces* sp. GSL17-111, sequencing and genome assembly were performed by Plasmidsaurus (1850 Millrace Drive, Suite 200 Eugene, Oregon 97 403). The GSL17-111 assembly consists of five contigs with an average coverage of 62×, an N50 value of 5 998 596, L50 value of 1, 97.6% genome completeness, and a contamination score of 0.71. BGCs were initially identified using antiSMASH v.7.0 [20], and clusters of interest were manually annotated and characterized using NCBI Blast/Blast+. BGCs identified in our GSL genomes that exhibited ≥85% similarity to characterized BGCs in the MIBiG v.3 database [44] were classified as “known” in our analyses.

### Comparative genomic and phylogenomic analyses of Great Salt Lake genomes

Phylogenomic analyses, including average nucleotide identity (ANI) and digital DNA–DNA hybridization (dDDH) were conducted using autoMLST [45] and the Type Strain Genome Server [46, 47]. Functional genomics were performed using the Anvi’o v.7.1 [48] suite of phylogenomic tools. Comparative genomic analyses were performed using the Anvi’o Pangenomic’s Workflow (https://merenlab.org/2016/11/08/pangenomics-v2/). For the soil- and marine-derived *Streptomyces* comparison, eight soil-derived *Streptomyces* spp. genomes were used (accession numbers: GCF_024752535.1, GCF_000717025.1, GCF_013307045.1, GCF_014655295.1, GCF_000010605.1, GCF_000739105.1, GCF_001886595.1, and GCF_014649995.1) and eight marine-derived *Streptomyces* spp. genomes were used (accession numbers: GCF_003443735.1, PRJNA175192, CP077658.1, GCF_014779715.1, CP054920, GCF_019303475.1, GCF_016803985.1, and GCF_004305975.1). For the closest related strain analysis for *Streptomyces* sp. GSL17-111 and *Saccharomonospora* GSL17-019, seven strains were used for each analysis (*Streptomyces* accession numbers: GCF_000718985.1, GCF_000813365.1, GCF_001493375.1, GCF_900110735.1, GCF_900111245.1, GCF_900114215.1, and GCF_900116145.1; *Saccharomonospora* accession numbers: GCF_000231035.2, GCF_000244955.1, GCF_000383775.1, GCF_000383795.1, GCF_000430445,1, GCF_000719975.1, and GCF_002077655.1). After the Anvi’o pangenomes were constructed, amino acid sequences of predicted genes unique to each GSL genome were extracted using the “anvi-get-sequences-for-gene-clusters” call, with a minimum functional homogeneity index of 0.75, and setting both “min-” and “max-num-genomes-gene-cluster-occurs” to 1 to extract only singleton genes. This resulted in a multi-FASTA file, from which sequences associated with GSL strains were extracted using a custom Python script. Sequences were then annotated using the eggNOG-mapper v.2 [49] web server using default settings. Distributions of genes and their associated Clusters of Orthologous Genes (COGs) categories were visualized in RStudio using the following packages: dplyr v.2.3.4, readr v.2.1.4, stringr v.1.5.0, ggplot2 v.3.4.2, purrr v.1.0.1, forcats v.1.0.0, and ggbreak v.0.1.2.

## Results

### Microbial diversity of Great Salt Lake sediments in the south arm

**Figure 1:**
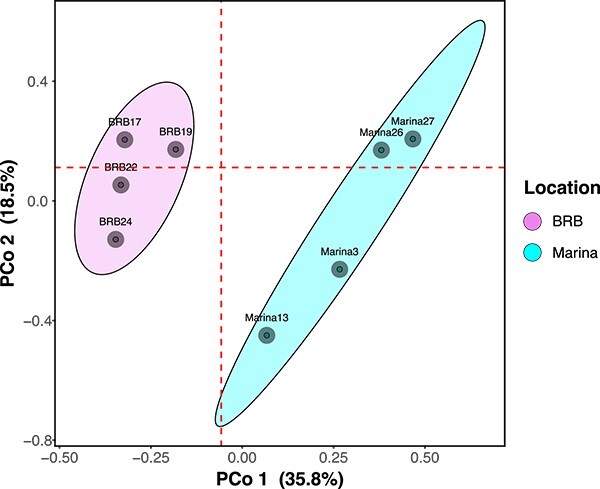
Bacterial community composition of sediment samples from BRB and the Marina regions, as measured by 16S rRNA gene amplicon sequencing; the PCoA plot illustrates overall community similarities and differences between four BRB and four Marina collection sites in the South Arm of GSL; dashed lines represent medians, and ellipses represent 75% confidence intervals around the samples from each region.

**Figure 2:**
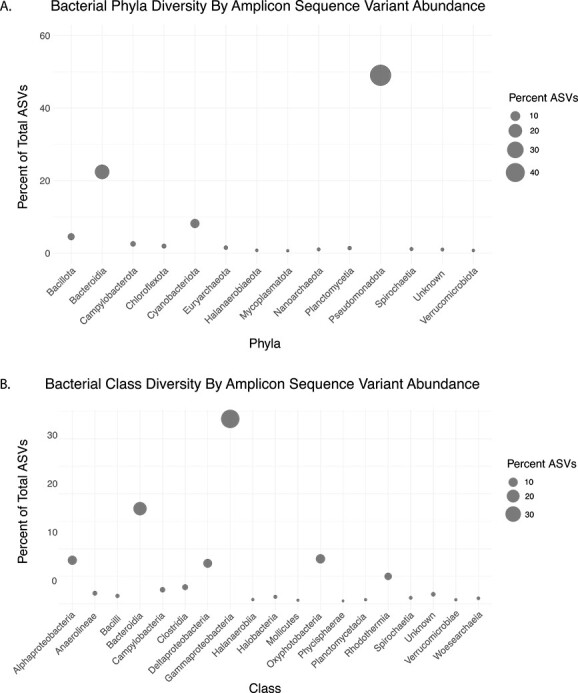
Diversity of GSL bacteria comprising at least 0.5% of total ASVs; **(**A) bubble chart illustrating diversity of 14 bacterial phyla identified by 16S ASV; (B) bubble chart illustrating diversity of 19 bacterial classes identified by 16S ASV; bacteria comprising <0.5% of total ASVs can be found in Fig. S1.

We collected eight sediment samples from two different geographic regions within the South Arm of GSL—BRB and the Marina, which are only ca. 180 m apart from each other. From each region, four sediment samples were collected and assessed for the presence of bacteria using 16S rRNA gene amplicon sequencing, which resulted in the identification of 748 251 ASVs (BRB = 441 628 and Marina = 306 623). Though we expected to see little variation in the bacterial community composition between our two geographically close collection sites, we found that the regions did exhibit significant differences in their taxonomic composition and structure (Fig. 1; Bray-Curtis, *P* < .05, PERMANOVA). Upon phylogenomic analysis, we found that the ASVs comprised 53 phyla and 421 genera (Fig. 2 and Fig. S1 and Table S1 and S2). We identified Pseudomonadota (49% of total ASVs) and Bacteroidota (22% of total ASVs) as the most abundant phyla (Fig. 2A and Fig. S1A, and Table S2), and Gammaproteobacteria (34% of total ASVs) and Bacteroidia (17% of total ASVs) as the most abundant classes (Fig. 2B and Fig. S1B). Among Gammaproteobacteria, *Sulfurimonas* and *Marinobacter* spp. were the most abundant (Table S1).

**Figure 3:**
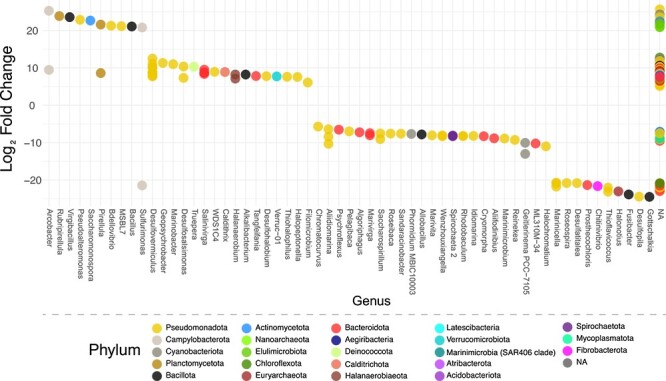
DESeq2 plot showing log_2_-fold change of phyla on the *y*-axis and genera on the *x*-axis, illustrating differential abundance of microorganisms between BRB and the Marina based on ASVs; a positive log_2_-fold-change indicates a significantly higher abundance at the Marina, while a negative log_2_-fold-change indicates a significantly higher abundance at BRB; analysis was generated using an adjusted *P*-value cutoff of <.01; NA/NA values represent unknown phylum/unknown genus and potentially represent uncharacterized bacteria.

Upon a closer inspection of our datasets, we found that the relative abundances of ASVs associated with specific organisms were more abundant in one of the two regions, either more abundant in BRB sediment or more abundant in Marina sediment. For example, ASVs associated with several Planctomycetota genera were found at significantly higher levels in the Marina sediment compared to BRB sediment (*Pirellula, P* = 3.68e-13, *P* = 2.9e-4; *Rubripirellula*, *P* = 1.25e-14, Wald test) (Fig. 3). In total, ASVs comprising 24 distinct phyla and 60 genera exhibited significantly different abundance between our two collection regions (Fig. 3 and Fig. S2). Interestingly, we observed differential abundances of ASVs representing two *Sulfurimonas* spp., with one found at much higher abundance in our BRB sample (*P* = 5.16e-11) and the other from the Marina sediment (*P* = 9.22e-11). Actinomycetota, which are common natural product producers, were found in both regions (0.31% of total ASVs), and an unknown *Saccharomonospora* species was found at a higher abundance in the Marina sediment compared to BRB (*P* = 5.24e-12) (Fig. 3 and Fig. S2).

### Taxonomic classification of Great Salt Lake Actinomycetota strains and comparative genomics studies

Using a culture-dependent approach, we isolated and sequenced three Actinomycetota strains, *Saccharomonospora* sp. GSL17-019, *Streptomyces* sp. GSL17-113, and *Streptomyces* sp. GSL17-111 from the sediment collected at the Marina. To determine the taxonomic position of these three strains, we conducted whole-genome phylogenomic analyses. ANI and dDDH analyses were conducted on each genome (Table S3) [45–47], and maximum-likelihood phylogenetic trees were assembled based on multilocus sequence analysis [45] (Figs S4–S6). Interestingly, while *Streptomyces* sp. GSL17–113 was closely related to *Streptomyces albus*, *Streptomyces* sp. GSL17-111 exhibited low relatedness to the reference *Streptomyces* strains. Indeed, ANI analysis revealed only 82.5% similarity to the closest related reference strain, *Streptomyces pini*. This falls far short of the 95%–96% species threshold. Additionally, GSL17-111 exhibited a dDDH range of 53.3%–58.8% identity (dDDH, d4) compared to its closest type strain, *Streptomyces chumphonensis* KK1-2, which is below the species threshold of 70%. Taken together, this strongly suggests that GSL17-111 represents a new *Streptomyces* species. *Saccharomonospora* sp. GSL17-019 also did not exhibit significant relatedness with any known *Saccharomonospora* species, with its closest related type strain ranging from 51.8% to 57.2% identity (dDDH, d4) and 93.5% ANI (Table S3). As these dDDH and ANI values are below the species threshold, we posit that strain GSL17-019 represents a new *Saccharomonospora* species that could be specific to GSL.

**Figure 4:**
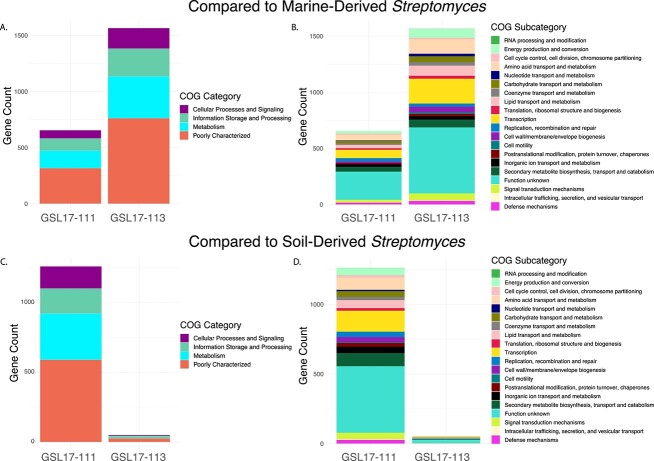
COG annotations of GSL *Streptomyces* with no homologs in soil- or marine-derived *Streptomyces* spp.; (A and B**)** genomic comparison of GSL *Streptomyces* spp. to marine-derived *Streptomyces* spp.; (A) functional classification of the 655 proteins encoded in *Streptomyces* sp. GSL17-111 and 1565 proteins identified in *Streptomyces* sp. GSL17-113; (B) distribution and classification by COG subcategories; (C and D**)** genomic comparison of GSL *Streptomyces* spp. to soil-derived *Streptomyces* spp.; (C) functional classification of the 1261 proteins encoded in *Streptomyces* sp. GSL17-111 and 50 proteins encoded in *Streptomyces* sp. GSL17-113; (D) distribution and classification by COG subcategories; a minimum functional homogeneity index cutoff of 0.75 was used to identify homologs in soil- or marine-derived *Streptomyces* spp.

To better understand the uniqueness of *Streptomyces* spp. GSL17-111 and GSL17-113, we independently carried out genomic comparisons of our GSL strains against eight soil- and eight marine-derived *Streptomyces* spp. using the Anvi’o [48] phylogenomic platform. Using a functional homogeneity index cutoff of 0.75, we identified and extracted all amino acid sequences with a minimum length of 40 residues unique to either GSL17-111 or GSL17-113. These sequences were then annotated using the COG database [50] (Fig. 4). When compared to marine-derived *Streptomyces* spp., we identified 799 genes unique to GSSL17-111. From the 799 identified genes, 655 sequences were annotated with a putative function using the eggNOG-mapper [49] annotation pipeline (Fig. 4A). Encoded gene products included transporters, regulators, and enzymes associated with primary and secondary metabolic processes, posttranslational modifications, and cell wall biogenesis. The most abundant proteins from each COG subcategory included those involved in signal transduction mechanisms, transcription, and secondary metabolite biosynthesis, transport, and catabolism (Fig. 4B). The majority of the annotated sequences, however, were predicted as hypothetical or proteins of unknown function, which was consistent for both *Streptomyces* spp. GSL17-111 and GSL17-113 across both comparative analyses. For the comparison of *Streptomyces* sp. GSL17-113 to marine-derived *Streptomyces* spp., 1776 unique genes were identified and of these 1565 were functionally annotated (Fig. 4A). The COG subcategories were similar to those identified in GSL17-111, although enzymes associated with transcription were far more abundant in GSL17-113 (Fig. 4B).

When compared to soil-derived *Streptomyces* spp., we identified only 89 genes unique to *Streptomyces* sp. GSL17-113. Of these, only 50 were functionally annotated using the eggnog mapper (Fig. 4C). This is unsurprising, given GSL17-113 exhibits significant similarity to the soil-dwelling *S. albus.* Although enzymes associated with transcription and replication, recombination, and repair were relatively abundant, the majority of the predicted gene products were characterized as hypothetical or proteins of unknown function (Fig. 4D). Unlike GSL17-113, when we compared *Streptomyces* sp. GSL17-111 to soil-derived *Streptomyces* spp., we identified 1475 unique genes. Of those identified, 1261 were functionally annotated (Fig. 4C). Similar to the marine comparison with GSL17-111, the most abundant COG subcategories included proteins involved in signal transduction mechanisms, transcription, and secondary metabolite biosynthesis, transport, and catabolism. Additional high-abundance subcategories included enzymes associated with cell wall/membrane/envelope biogenesis; replication, recombination, and repair; amino acid transport and metabolism; and inorganic ion transport and metabolism (Fig. 4D). Taken together, these results suggest that *Streptomyces* sp. GSL17-113 is more closely related to soil-derived *Streptomyces* spp., whereas *Streptomyces* sp. GSL17-111 is more closely related to marine-derived *Streptomyces* spp. However, it should be noted that *Streptomyces* sp. GSLl17-111 is indeed quite unique and exhibited an overall low genomic similarity to both marine-derived and terrestrial *Streptomyces* spp. (Fig. 4 and Fig. S6 and Table S3).

**Figure 5:**
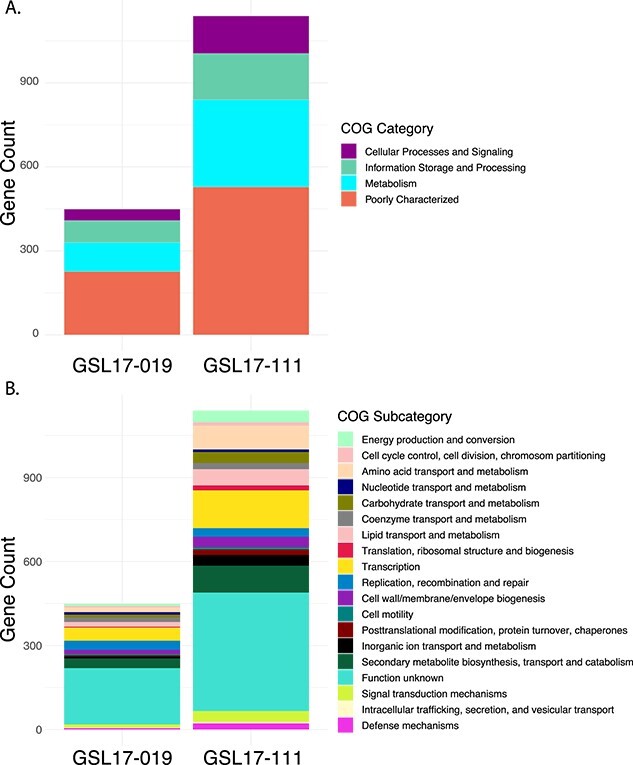
COG annotations encoded by GSL *Streptomyces* found to have no homologs in their closest-related reference strains; (A) functional classification of the 1139 proteins encoded in *Streptomyces* sp. GSL17-111 and 449 proteins identified in *Saccharomonospora* sp. GSL17-019; (B) distribution and classification by COG subcategories; taxonomic position was determined using the autoMLST [45] phylogenomic pipeline and a phylogenetic tree was constructed for each strain; based on the maximum-likelihood trees (Figs S4 and S6), the closest seven strains from each analysis were used for comparative genomics.

To further evaluate whether *Streptomyces* sp. GSL17-111 and *Saccharomonospora* sp. GSL17-019 represent new species, we again employed a comparative genomics approach. Based on the maximum-likelihood phylogenetic trees constructed using multilocus sequence analysis, we chose seven of the most closely related strains for both GSL17-111 and GSL17-019 for additional comparisons. For GSL17-111, all seven strains were *Streptomyces* spp., while the seven closest related strains to GSL17-019 were either *Saccharomonospora* or *Actinopolyspora* spp. and included the closest related strain identified in the dDDH analysis (*Saccharomonospora iraqiensis* subsp. *paurometabolica* YIM90007, accession number GCF_000231035.2). Again, we identified and extracted all amino acid sequences with a minimum length of 40 residues unique to either GSL17-111 or GSL17-019 using a functional homogeneity index cutoff of 0.75 and used eggNOG-mapper for putative annotations (Fig. 5). From this analysis, we identified 1342 genes unique to *Streptomyces* sp. GSL17-111. Of these, 1139 were functionally annotated (Fig. 5A). Aside from the uncharacterized/hypothetical categories, genes encoding proteins associated with metabolism represented the most abundant COG category. This included enzymes involved with secondary metabolite biosynthesis, inorganic ion transport, and amino acid transport and metabolism. Enzymes associated with transcription represent the most abundant COG subcategory and proteins involved with cellular processes, and signal transduction mechanisms, cell wall/membrane/envelope biogenesis, and defense mechanism represent the most abundant subcategories (Fig. 5B). Proteins associated with cell cycle control, cell division and chromosomal partitioning, cell motility, carbohydrate transport and metabolism, posttranslational modification, protein turnover, and chaperones were also identified, illustrating a range of enzymes involved with many important cellular functions that may be necessary to thrive in the extreme environment of GSL.

When compared to the closest related reference *Saccharomonospora* spp., we identified 636 genes unique to *Saccharomonospora* sp. GSL17-019. Of these, 449 sequences were annotated with a putative COG function (Fig. 5A). Aside from genes encoding hypotheticals and proteins with unknown function, the most abundant category was associated with metabolism and included proteins involved with secondary metabolite biosynthesis, coenzyme transport and metabolism, and lipid transport and metabolism. Genes encoding enzymes associated with transcription, replication, recombination, and repair, as well as proteins involved with cell wall/membrane/envelope biogenesis and signal transduction mechanisms were also identified (Fig. 5B). Given the large number of unique genes, even when compared to the closest related reference strains, these data further support the uniqueness of *Streptomyces* sp. GSL17-111 and *Saccharomonospora* sp. GSL17-019.

### Natural product potential of Great Salt Lake bacteria

**Figure 6:**
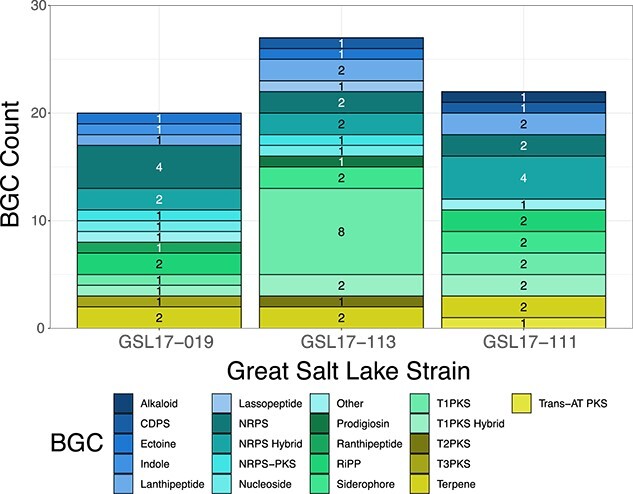
Number and type of BGCs identified in *Saccharomonospora* sp. GSL17-019, *Streptomyces* sp. GSL17-113, and *Streptomyces* sp. GSL17-111; abbreviations: cyclodipeptide synthase, CDPS; nonribosomal peptide synthetase, NRPS; polyketide synthase, PKS; ribosomally synthesized and post-translationally modified peptide, RiPP; acyltransferase, AT; Type I polyketide synthase, T1PKS; Type II polyketide synthase, T2PKS; Type III polyketide synthase, T3PKS.

**Figure 7:**
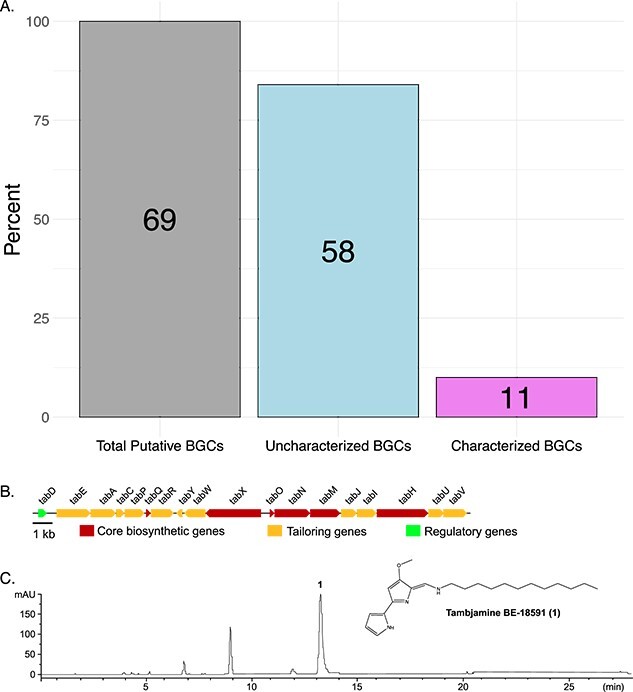
Natural product potential of *Saccharomonospora* sp. GSL17-019, *Streptomyces* sp. GSL17-113, and *Streptomyces* sp. GSL17-111; (A) a total of 69 BGCs were identified from the three GSL strains; of those, 11 were strongly associated with characterized BGCs deposited in MIBiG v.3 (≥85% predicted similarity) and included BGCs responsible for ectoine, coprisamide, xenematide, desferriosamine, tambjamine, geosmin, isorenieratene, and piericidine biosynthesis; the remaining 58 BGCs were not strongly associated with known gene clusters; BGC-associated compounds were identified through gene cluster comparison using both antiSMASH v.7.0 and manual annotation; (B) organization of the tambjamine BGC identified in *Streptomyces* sp. GSL17-113; (C) high-performance liquid chromatography analysis (390 nm) of GSL17-113 and identification of tambjamine BE-18591 (1).

We investigated the potential for specialized metabolite production by our sequenced isolates through the identification and annotation of natural product BGCs encoded in each genome (Fig. 6 and Tables S4–S6). We identified 20 putative BGCs in *Saccharomonospora* sp. GSL17-019 (Table S4) and 27 putative BGCs in *Streptomyces* sp. GSL17–113 (Table S5), representing 19 different BGC classes. These classes included more common polyketide synthase and nonribosomal peptide synthetase-containing clusters as well as less common ribosomally synthesized and post-translationally modified peptide clusters predicted to produce lassopeptide [51] and ranthipeptide [51, 52] natural products (Fig. 6). From *Streptomyces* sp. GSL17-111, we identified 22 BGCs (Fig. 6 and Table S6). Of the combined BGCs identified, only 11 strongly correlated (≥85% predicted similarity) to characterized clusters in public databases that have been associated with specific natural products (Fig. 7A). To better assess the natural product potential of our GSL isolates, *Streptomyces* sp. GSL17-113 was subjected to small-scale cultivation studies. From the culture extract, we identified tambjamine BE-18591 (Fig. 7B and C and Figs S7 and S8 and Table S7). Tambjamine BE-18591 was originally reported from *Streptomyces* sp. BA18591, a plant-derived isolate collected in Japan [53, 54]. The tambjamines possess antimicrobial and cytotoxic activities [53, 55], and tambjamine BE-18591 has been reported to possess antimicrobial activity against both fungi and bacteria, including *Candida albicans*, *Malassezia furfur*, *E. coli*, and *Staphylococcus aureus* [53, 55]. Further, tambjamine BE-18591 displays broad antitumor activity against leukemia, melanoma, colorectal, and glioblastoma cell lines [55], as well as inhibition of immunoproliferation and gastritis in rabbits [56]. Our genomic analysis also confirmed the presence of the tambjamine BE-18591 BGC in *Streptomyces* sp. GSL17-113 (Fig. 7B and Tables S5 and S8), emphasizing the importance and utility of genome mining when prioritizing strains for downstream fermentation studies and aiding in the dereplication process. Though our initial fermentation study resulted in the isolation of a known compound, >80% of the identified gene clusters (>50 BGCs) annotated in our three datasets had weak to no similarity with characterized BGCs, indicating that the corresponding biosynthetic machinery could be synthesizing new natural product scaffolds.

## Discussion

In this study, we have highlighted the diversity of GSL’s unique microbiome, including the genomic characterization of a potentially new *Saccharomonospora* and *Streptomyces* species, and emphasized the natural product potential of GSL bacteria to produce bioactive compounds. Previously, we isolated the bonnevillamides from a GSL bacterium, *Streptomyces* sp. GSL-6B. The bonnevillamides are linear heptapeptides containing a distinctive 3-(3,5-dichloro-4-hydroxyphenyl)-2-methoxypropenoic acid moiety, and bonnevillamide A harbors an additional unprecedented 4-methyl-azetidine-2-carboxylic acid methyl ester chemical motif [57, 58]. We also reported on the isolation and structure elucidation of the salinipeptins, ribosomally synthesized and post-translationally modified peptides containing rare d-amino acids, a highly functionalized N-terminus, and a C-terminal aminovinyl-cysteine residue [59]. Importantly, salinipeptin A displayed moderate activity against Group A *Streptococcus pyogenes* as well as glioblastoma and colon cancer cell lines. Although tambjamine BE-18591 is a known natural product, its isolation from *Streptomyces* sp. GSL17-113 represents an additional bioactive natural product isolated from a GSL bacteria. Importantly, when we queried the tambjamine BE-18591 BGC through the NCBI database, only three *Streptomyces* spp. displayed query coverage above 62% (Table S9), suggesting that tambjamine BE-18591 is an uncommon natural product. When taken with the comparative and phylogenomic analyses, our research supports GSL as an underexplored environment harboring uncharacterized bacteria potentially rich in bioactive natural products. Additional fermentation studies with both *Streptomyces* sp. GSL17-111 and *Saccharomonospora* sp. GSL17-019 are currently underway to further characterize the natural product potential of these strains.

In parallel with the isolation and characterization of novel natural products, we intend to explore potential resistance mechanisms utilized by GSL microbes. As GSL is a terminal lake and serves as the endpoint for wastewater treatment runoff, we anticipate it to be a significant source of antibiotic-resistant pathogenic microbes, especially as the environment can serve as a reservoir for antibiotic-resistant bacteria [60–65]. Furthermore, from an ecological standpoint, as GSL water levels fluctuate, it is important that we continue to investigate the Lake’s microbiome and how the changing water levels, drought, and inflow affect microbial diversity. From this study, we not only provide an initial insight into the diversity of GSL sediment-derived bacteria but also provide the first genomic characterization of Actinomycetota isolated from GSL. This is of particular interest to our group, as Actinomycetota are the dominant producers of therapeutic compounds essential for human health [63, 66–68].

Bioprospecting of GSL bacteria led to the identification of the bonnevillamides, salinipeptins, and in this study, the antitumor and antibiotic compound, tambjamine BE-18591. Given the numerous unidentified BGCs (Fig. 7A and Tables S4–S6), it is evident that GSL represents a largely untapped resource of natural products. Though our primary interest is in characterizing known natural product producing microbes, the high number of unknown phylum/unknown genus values we observed in our sediment samples (Fig. 3) suggests that GSL is home to many as-of-yet characterized bacteria. Though several common phyla identified in this study were similar to those identified in a similar report investigating marine sediment-derived bacteria [69], there were several differences in the microbial composition of GSL sediment. The most abundant phyla identified from marine sediment, in the order of abundance, included Pseudomonodota, Cyanobacteriota, unclassified species, Bacteroidia, and Desulfobacteria [69]. In our study, the most abundant phyla identified included Pseudomonadota, Bacteroidia, Cyanobacteriota, Bacillota, and Campylobacterota (Fig. 2A and Fig. S1A and Table S2), indicating a differential microbial composition compared to marine sediment and further supporting GSL is unique compared to marine or terrestrial environments. GSL’s uniqueness is additionally emphasized by the discovery and genomic characterization of the potentially new *Streptomyces* sp. GSL17-111 and *Saccharomonospora* sp. GSL17-019 isolated from the GSL sediment. Taken together, our results give an overview of GSL’s large microbial and chemical diversity as well as differences in microbial populations between geographically close regions and revealed GSL’s potential to harbor new species of natural product–producing bacteria.

## Supplementary Material

GSL_SI_REVISED_final_feb_ycae029

## Data Availability

All relevant code and data, including genes identified in the comparative genomic analysis, are available at https://github.com/ERBringHorvath/Great-Salt-Lake-Microbial-Diversity. 16S amplicon sequences are available under BioProject accession number PRJNA975952. The genomes of *Streptomyces* sp. GSL17-113 and *Saccharomonospora* sp. GSL17-019 are deposited under BioProject accession number PRJNA1066849. The genome of *Streptomyces* sp. GSL17-111 is deposited under BioProject accession number PRJNA1077303. The tambjamine BE-18591 biosynthetic cluster from *Streptomyces* sp. GSL17-113 is deposited under the GenBank accession number PP179500.
