# Supplementary material for: Bacterial diversity and chemical ecology of natural product–producing bacteria from Great Salt Lake sediment
Source: ISME Commun. 2024 Mar 4;4(1):ycae029. doi: 10.1093/ismeco/ycae029 (PMC10960970; doi:10.1093/ismeco/ycae029)
Supplement: GSL_SI_REVISED_final_feb_ycae029 [file gsl_si_revised_final_feb_ycae029.docx]

**Supplementary Information**

**Bacterial Diversity and Chemical Ecology of Natural Product-Producing Bacteria from Great Salt Lake Sediment**

**Author Information**

Elijah R. Bring Horvath,^1,2,3^ William J. Brazelton,^2^ Min Cheol Kim,^4^ Reiko Cullum,^4^ Matthew A. Mulvey,^2,5^ William Fenical,^4,6,7^ Jaclyn M. Winter^1,^*

^1^Department of Pharmacology and Toxicology, University of Utah, Salt Lake City, Utah, United States; ^2^School of Biological Sciences, University of Utah, Salt Lake City, Utah, United States; ^3^Department of Medicinal Chemistry, University of Utah, Salt Lake City, Utah, United States; ^4^Center for Marine Biotechnology and Biomedicine, Scripps Institution of Oceanography, University of California at San Diego, San Diego, California, United States; ^5^Henry Eyring Center for Cell and Genome Science, University of Utah, Salt Lake City, Utah, United States; ^6^Skaggs School of Pharmacy and Pharmaceutical Sciences, University of California at San Diego, San Diego, California, United States; ^7^Moores Comprehensive Cancer Center, University of California at San Diego, San Diego, California, United States.

^*^Corresponding author: Jaclyn M. Winter (jaclyn.winter@utah.edu)

**Table of Contents**

**Supplementary Tables**

Table S1: DADA2 output and taxonomic classification of amplicon sequence variants **S3**

Table S2: Diversity and amplicon sequence variant abundance of GSL microbes by phyla and class. **S8**

Table S3: ANI and dDDH results for *Saccharomonospora* sp. GSL17-019, *Streptomyces* sp. GSL17-113, GSL17-111 **S10**

Table S4: antiSMASH results of *Saccharomonospora* sp. GSL17-019 **S14**

Table S5: antiSMASH results of *Streptomyces* sp. GSL17-113 **S15**

Table S6: antiSMASH results of *Streptomyces* sp. GSL17-111 **S16**

Table S7: ^1^H NMR assignment of tambjamine BE-18591 **S17**

Table S8: Putative tambjamine BE-18591 biosynthetic cluster in *Streptomyces* sp. GSL17-113 **S20**

Table S9: Strains predicted to encode the tambjamine BE-18591 BGC identified in *Streptomyces* sp. GSL17-113 **S21**

**Supplementary Figures**

Figure S1: Diversity of Great Salt Lake bacteria by amplicon sequence variant (ASV) abundance **S4**

Figure S2: Differential abundance of ASVs associated with Actinomycetota and Enterobacteriaceae **S5**

Figure S3: DESeq2 analysis of differentially abundant ASVs **S6**

Figure S4: Maximum-likelihood phylogenetic tree of *Saccharomonospora* sp. GSL17-019 **S11**

Figure S5: Maximum-likelihood phylogenetic tree of *Streptomyces* sp. GSL17-113 **S12**

Figure S6: Maximum-likelihood phylogenetic tree of *Streptomyces* sp. GSL17-111 **S13**

Figure S7: ^1^H NMR spectrum of tambjamine BE-18591 isolated from *Streptomyces* sp. GSL17-113 **S18**

Figure S8. UV profile and HR-ESI-MS of tambjamine BE-18591 **S19**

**Supplemental References S22**

**Table S1.** DADA2 output and taxonomic classification of amplicon sequence variants.

Due to the size of this table, it is available as a separate downloadable CSV file.


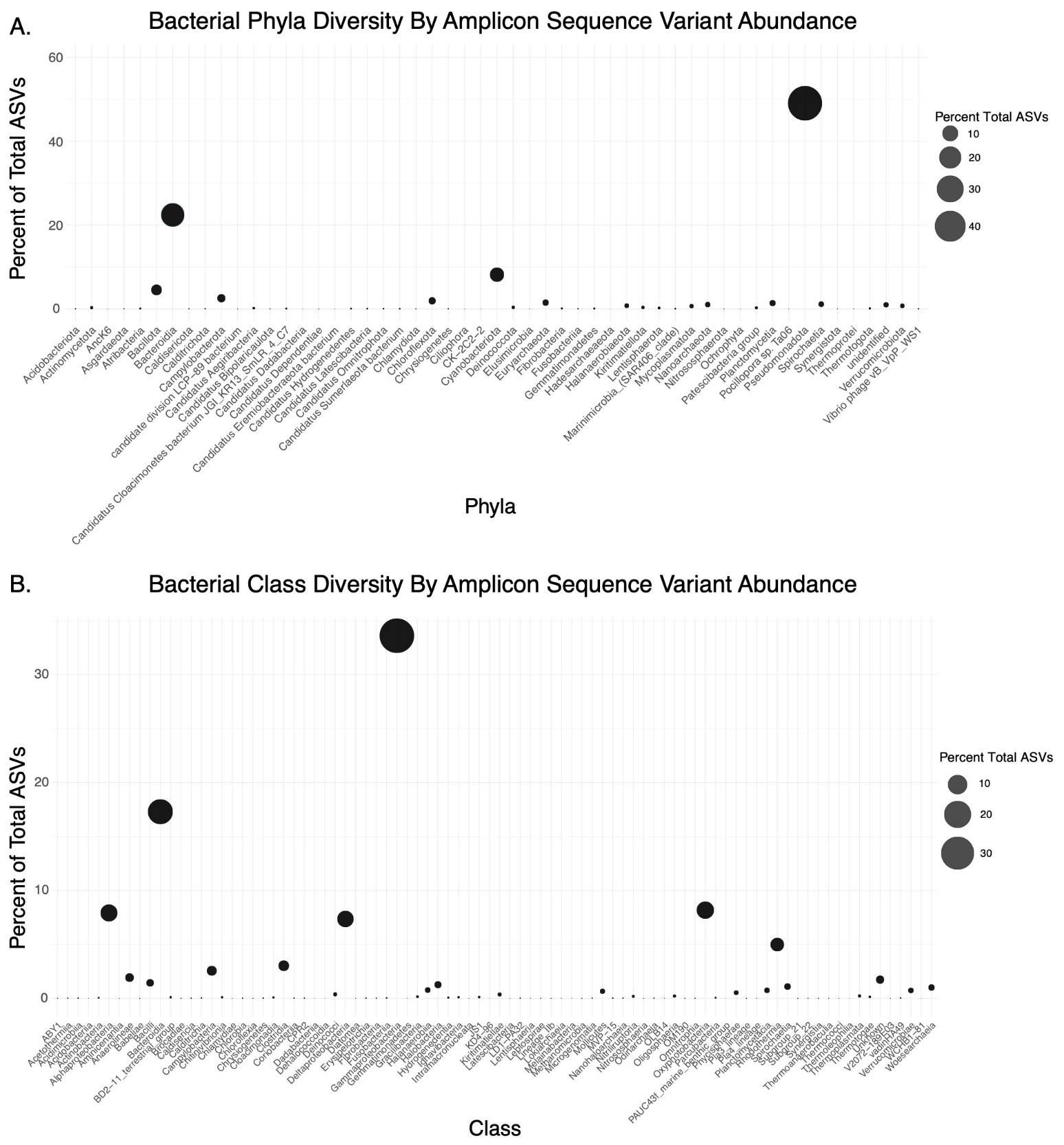


**Figure S1.** Diversity of Great Salt Lake bacteria by amplicon sequence variant (ASV) abundance. **A.** Bubble chart illustrating diversity of 53 bacterial phyla identified by 16S ASV. **B.** Bubble chart illustrating diversity of 86 bacterial classes identified by 16S ASV.


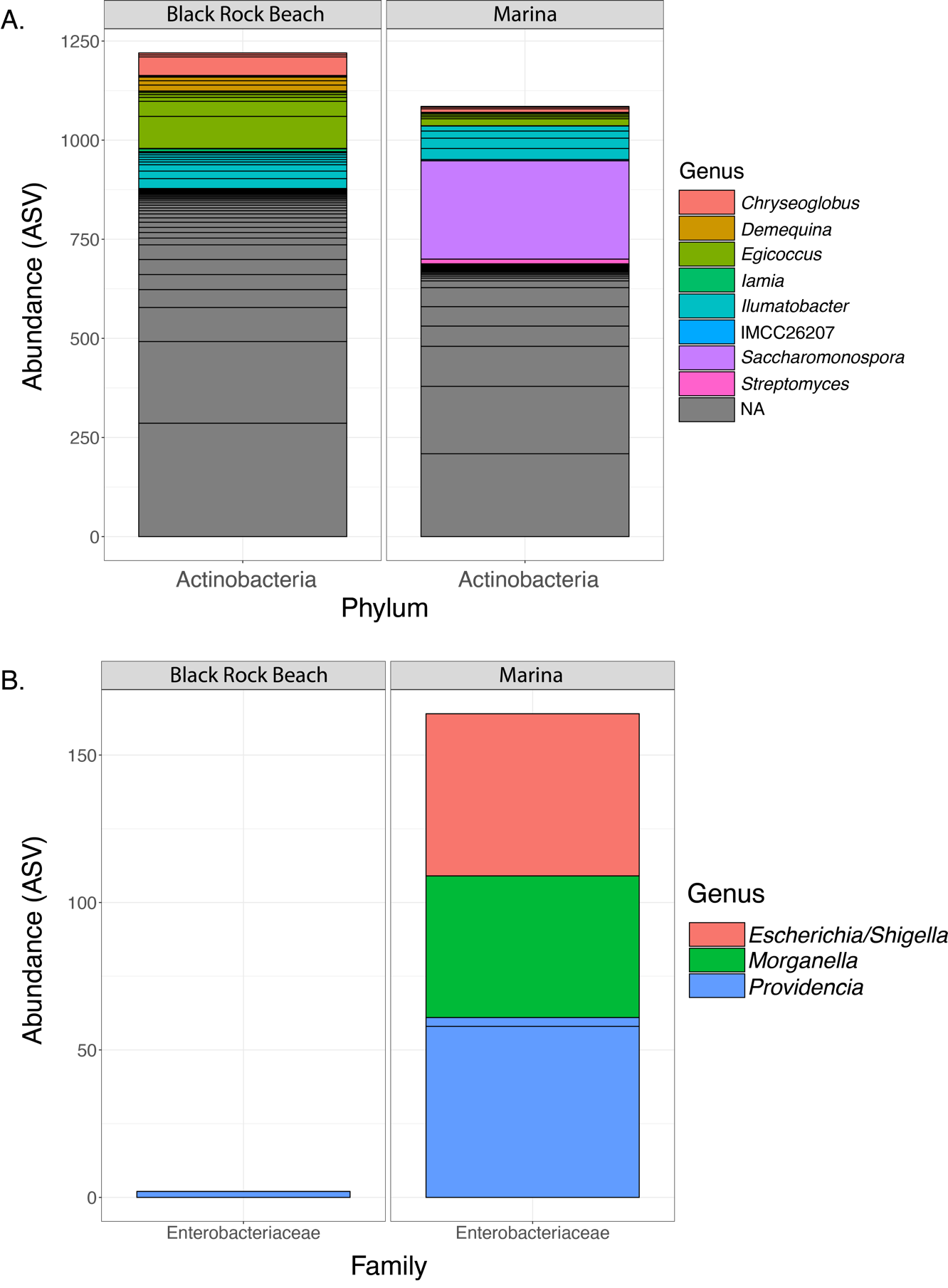


**Figure S2**. Differential abundance of amplicon sequence variants (ASVs) categorized to represent Actinomycetota and Enterobacteriaceae between Black Rock Beach and Marina samples. **A.** Colors represent Actinomycetota genera identified in our analysis. Of these, ASVs categorized to represent *Saccharomonospora* were found at significantly higher abundance in the Marina vs BRB. NA values represent unknown genera. **B.** Differential abundance of Enterobacteriacaea family organisms between BRB and Marina.

**
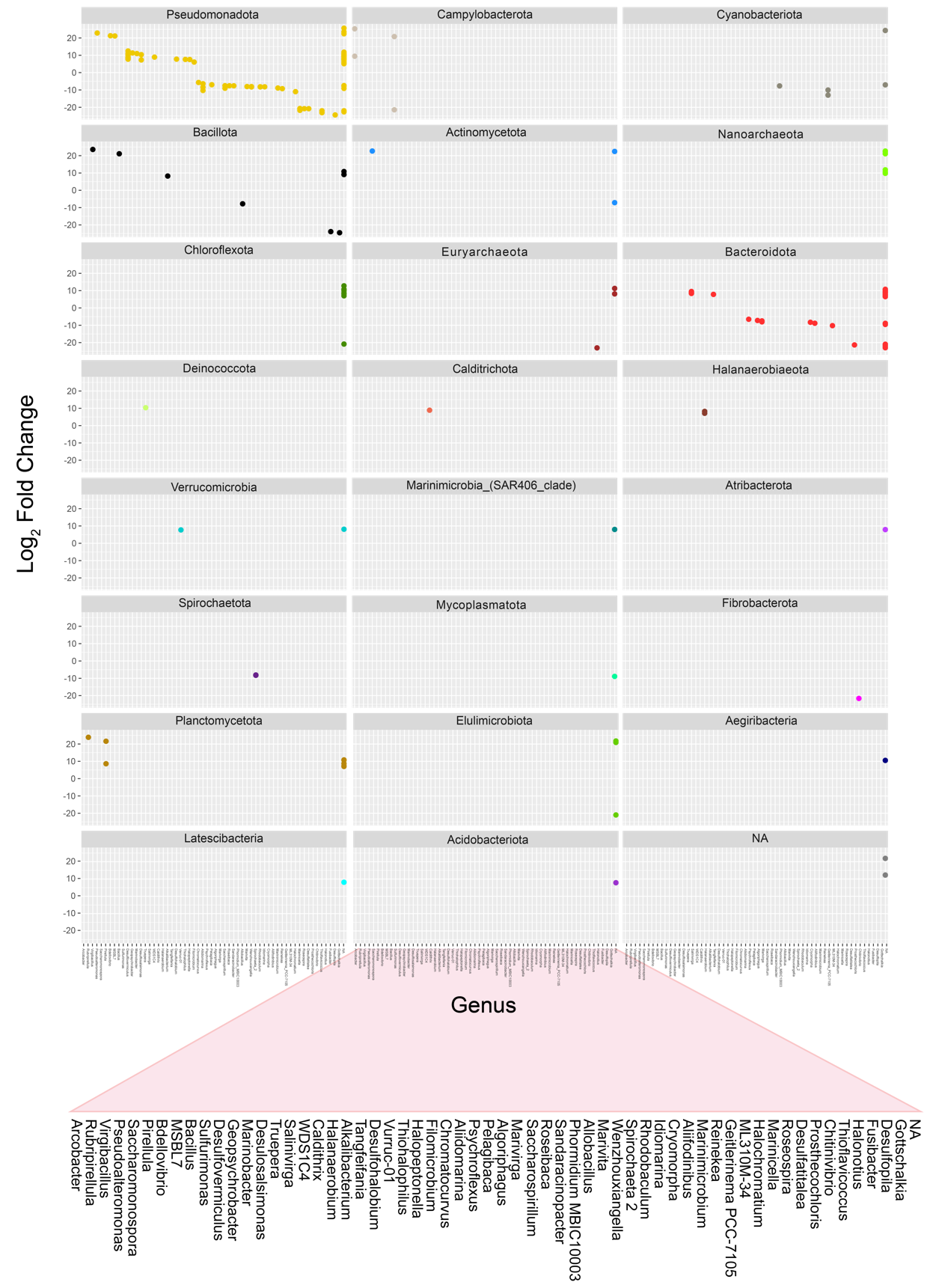
**

**Figure S3**. Facet wrapped DESeq2 [1] plot showing phyla on the y-axis and genera on the x-axis illustrating differential abundance of microbes between Black Rock Beach and the Marina based on amplicon sequence variants (ASV). Phyla are displayed on the y-axis and genera on the x-axis. A positive log2 fold-change indicates a significantly higher abundance at the Marina, while a negative log2 fold-change indicates significantly higher abundance at Black Rock Beach. Analysis was generated using an alpha value cutoff of < 0.01. NA/NA values represent unknown phylum/unknown genus, and potentially represent uncharacterized microbes. This is an expansion of Figure 3, with each organism assigned its own tile. Each tile is titled by organism phylum. The zoomed in section offers an enlarged view of the x-axis labels, which is the same between all tiles.

**Table S2.** Diversity and amplicon sequence variant (ASV) abundance of GSL microbes by phyla and class.

| **Phylum** | **Percent of total ASVs** | **Class** | **Percent of total ASVs** | **Class** | **Percent of total ASVs** |
| --- | --- | --- | --- | --- | --- |
| Pseudomonadota | 49.05 | Gammaproteobacteria | 33.61 | Omnitrophia | 0.0143 |
| Bacteroidia | 22.42 | Bacteroidia | 17.30 | Brocadiae | 0.0142 |
| Cyanobacteriota | 8.18 | Oxyphotobacteria | 8.18 | OM190 | 0.0127 |
| Bacillota | 4.54 | Alphaproteobacteria | 7.92 | Subgroup_22 | 0.0126 |
| Campylobacterota | 2.56 | Deltaproteobacteria | 7.36 | Leptospirae | 0.0119 |
| Chloroflexota | 1.94 | Rhodothermia | 4.99 | Synergistia | 0.0119 |
| Euryarchaeota | 1.51 | Clostridia | 3.03 | ABY1 | 0.0111 |
| Planctomycetota | 1.40 | Campylobacteria | 2.56 | Subgroup_21 | 0.0102 |
| Spirochaetia | 1.14 | Anaerolineae | 1.93 | Pla3_lineage | 0.0083 |
| Nanoarchaeota | 1.04 | Unknown | 1.74 | Chloroflexia | 0.0072 |
| unidentified | 1.00 | Bacilli | 1.44 | Pla4_lineage | 0.0063 |
| Halanaerobiaeota | 0.78 | Halobacteria | 1.27 | Lokiarchaeia | 0.0056 |
| Verrucomicrobiota | 0.74 | Spirochaetia | 1.10 | Nitrososphaeria | 0.0049 |
| Mycoplasmatota | 0.66 | Woesearchaeia | 1.01 | Thermoanaerobaculia | 0.0049 |
| Deinococcota | 0.38 | Halanaerobiia | 0.78 | Lineage_IIb | 0.0048 |
| Kiritimatiellota | 0.37 | Planctomycetacia | 0.75 | Latescibacteria | 0.0041 |
| Actinomycetota | 0.31 | Verrucomicrobiae | 0.74 | Chlamydiae | 0.0037 |
| Patescibacteria group | 0.28 | Mollicutes | 0.66 | Aminicenantia | 0.0029 |
| Lentisphaerota | 0.23 | Phycisphaerae | 0.53 | V2072-189E03 | 0.0029 |
| Candidatus Aegiribacteria | 0.20 | Deinococci | 0.38 | Lentisphaeria | 0.0027 |
| Thermotogota | 0.14 | Kiritimatiellae | 0.37 | Diatomea | 0.0021 |
| Fibrobacteria | 0.13 | Thermoplasmata | 0.23 | Thermococci | 0.0021 |
| Atribacteria | 0.12 | Oligosphaeria | 0.22 | Intramacronucleata | 0.0016 |
| Gemmatimonadetes | 0.11 | Nitriliruptoria | 0.19 | Melainabacteria | 0.0016 |
| Candidatus Cloacimonetes bacterium JGI_KR13_SmLR_4_C7 | 0.10 | Gracilibacteria | 0.16 | OLB14 | 0.0012 |
| Candidatus Hydrogenedentes | 0.08 | Thermotogae | 0.14 | Acidobacteriia | 0.0009 |
| Candidatus Latescibacteria | 0.07 | JS1 | 0.12 | Gemmatimonadetes | 0.0008 |
| Marinimicrobia_(SAR406_clade) | 0.07 | Chitinivibrionia | 0.11 | Dehalococcoidia | 0.0007 |
| Candidatus Sumerlaeota bacterium | 0.06 | Ignavibacteria | 0.11 | Thermoleophilia | 0.0005 |
| Candidatus Omnitrophota | 0.06 | BD2-11_terrestrial_group | 0.11 | WCHB1-81 | 0.0005 |
| Vibrio phage vB_VpP_WS1 | 0.05 | Cloacimonadia | 0.10 | Dadabacteriia | 0.0004 |
| Calditrichota | 0.04 | Hydrogenedentia | 0.08 | PAUC43f_marine_benthic_group | 0.0004 |
| Fusobacteriia | 0.04 | Parcubacteria | 0.07 | KD4-96 | 0.0003 |
| Chrysiogenetes | 0.03 | Actinobacteria | 0.06 |  |  |
| Acidobacteriota | 0.03 | vadinHA49 | 0.04 |  |  |
| Chlamydiota | 0.02 | Calditrichia | 0.04 |  |  |
| Asgardaeota | 0.02 | Fusobacteriia | 0.04 |  |  |
| Caldisericota | 0.02 | Nanohaloarchaeia | 0.03 |  |  |
| Candidatus Bipolaricaulota | 0.02 | Chrysiogenetes | 0.03 |  |  |
| Candidatus Dependentiae | 0.01 | Acidimicrobiia | 0.03 |  |  |
| Synergistota | 0.01 | MVP-15 | 0.03 |  |  |
| Thermoprotei | 0.01 | Coriobacteriia | 0.03 |  |  |
| candidate division LCP-89 bacterium | 0.01 | Erysipelotrichia | 0.03 |  |  |
| Nitrososphaerota | 0.0049 | Microgenomatia | 0.02 |  |  |
| Elusimicrobia | 0.0048 | Chlorobia | 0.02 |  |  |
| Ochrophyta | 0.0021 | LD1-PA32 | 0.02 |  |  |
| Ciliophora | 0.0016 | Caldisericia | 0.02 |  |  |
| CK-2C2-2 | 0.0013 | Fibrobacteria | 0.02 |  |  |
| AncK6 | 0.0012 | CPR2 | 0.02 |  |  |
| Candidatus Eremiobacteraeota bacterium | 0.0011 | Odinarchaeia | 0.02 |  |  |
| Candidatus Dadabacteria | 0.0004 | Acetothermiia | 0.02 |  |  |
| Hadesarchaeaeota | 0.0004 | Babeliae | 0.01 |  |  |
| Pocillopora sp. Ta06 | 0.0004 | Methanomicrobia | 0.01 |  |  |

**Table S3**. Average nucleotide identity and digital DNA-DNA hybridization analysis of *Streptomyces* sp. GSL17-113 and *Saccharomonospora* sp. GSL17-019. ANI = average nucleotide identity. dDDH = digital DNA-DNA hybridization. C.I. = confidence interval.

| **GSL Strain** | **GSL17-113** | **GSL17-019** | **GSL17-111** |
| --- | --- | --- | --- |
| **Closest Reference Strain (ANI)** | *Streptomyces albus*, subsp. *albus* | [*Actinopolyspora*]_*iraqiensis*_IQ-H1 | *Streptomyces pini* |
| **Reference Strain Assembly (ANI)** | GCF_000725885 | GCF_000430445 | GCF_900114215 |
| **Estimated ANI** | 0.993 | 0.935 | 0.825 |
| **Reference Strain (dDDH)** | *Streptomyces albus* NBRC 13014 | *Saccharomonospora iraqiensis* subsp. *paurometabolica* YIM90007 | *Streptomyces chumphonensis* KK1-2 |
| **dDDH (d0, in %)** | 90.5 | 42.5 | 78.6 |
| **C.I. (d0, in %)** | [87.3 - 92.9] | 39.1 - 45.9 | [74.7 - 82.1] |
| **dDDH (d4, in %)** | 95.7 | 54.5 | 56 |
| **C.I. (d4, in %)** | [94.2 - 96.8] | 51.8 - 57.2 | [53.3 - 58.8] |
| **dDDH (d6, in %)** | 93.7 | 43.9 | 76.4 |
| **C.I. (d6, in %)** | [91.5 - 95.3] | 40.9 - 47.0 | [72.9 - 79.5] |
| **Consensus** | *Streptomyces albus* | N/A | N/A |


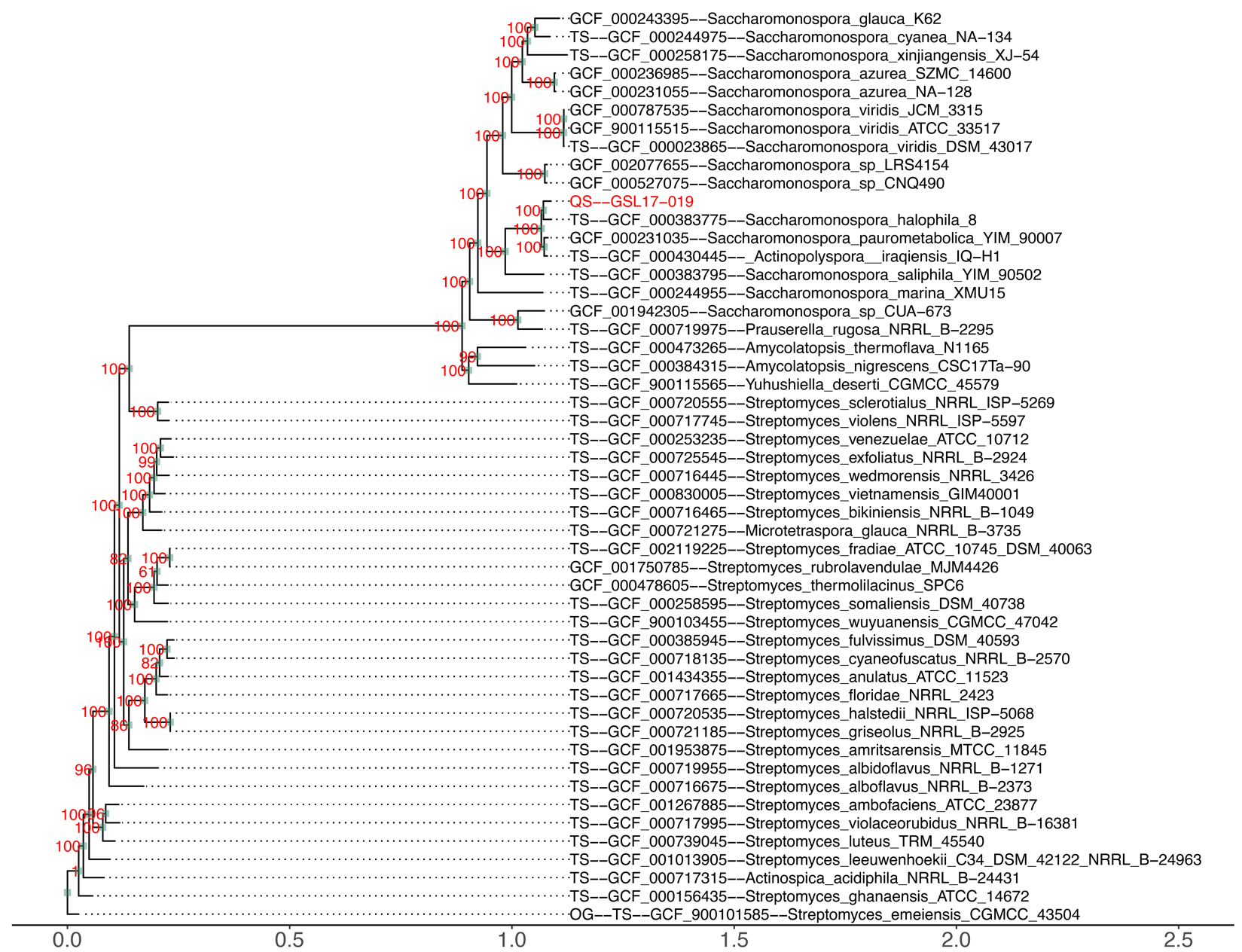


**Figure S4**. Maximum-likelihood phylogenetic tree of *Saccharomonospora* sp. GSL17-019 with reference strains. Phylogenetic analysis was performed using autoMLST with IQ-TREE [2] Ultrafast Bootstrap analysis using 1000 replicates. *Streptomyces emeiensis* CGMCC 43054 was assigned as the outgroup.

**
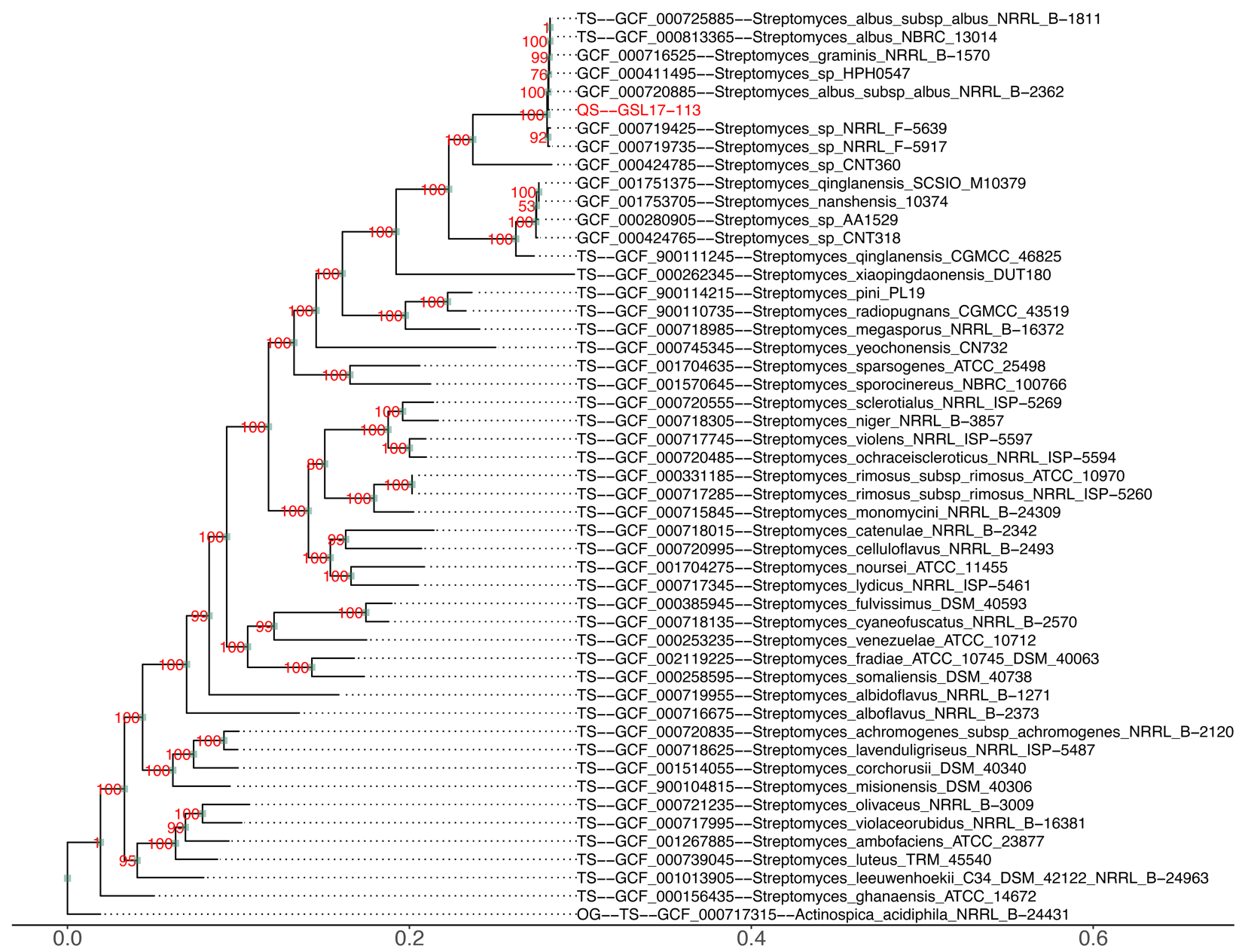
**

**Figure S5**. Maximum-likelihood phylogenetic tree of *Streptomyces* sp. GSL17-113 with reference strains. Phylogenetic analysis was performed using autoMLST with IQ-TREE [2] Ultrafast Bootstrap analysis using 1000 replicates. *Actinocpica acidiphila* NRRL B-24431 was assigned as the outgroup.

**
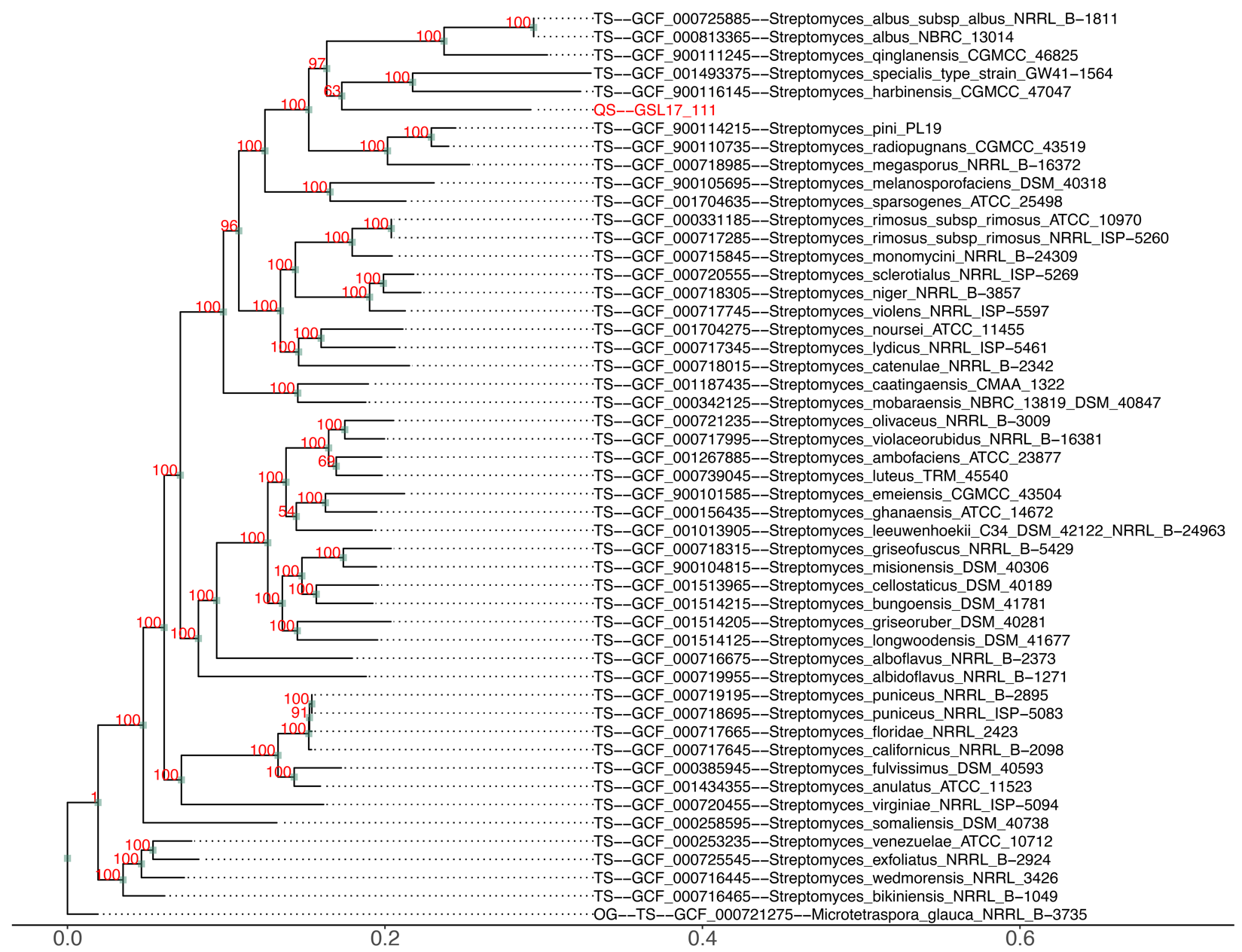
**

**Figure S6.** Maximum-likelihood phylogenetic tree of *Streptomyces* sp. GSL17-111 with reference strains. Phylogenetic analysis was performed using autoMLST with IQ-TREE [2] Ultrafast Bootstrap analysis using 1000 replicates. *Microtetraspora glauca* NRRL B-3725 was assigned as the outgroup.

**Table S4**. antiSMASH [3] output table of predicted biosynthetic gene clusters identified *Saccharomonospora* sp. GSL17-019 (BioProject accession number PRJNA1066849 and Biosample number SAMN39507642).

| **Region** | **Type** | **From** | **To** | **Most similar known cluster** | **Similarity** |
| --- | --- | --- | --- | --- | --- |
| Region 1.1 | indole | 340,734 | 361,840 | fortimicin | 9% |
| Region 1.2 | ectoine | 686,756 | 697,157 | ectoine | 100% |
| Region 1.3 | arylpolyene,ladderane,NRPS | 942,573 | 1,012,591 | coprisamides | 86% |
| Region 2.1 | other,NRPS | 16,861 | 67,002 | lidamycin | 15% |
| Region 2.2 | ranthipeptide | 154,725 | 176,239 | N/A | N/A |
| Region 2.3 | T1PKS | 714,046 | 785,157 | kendomycin B | 15% |
| Region 2.4 | T1PKS,terpene | 850,069 | 909,489 | maduropeptin | 34% |
| Region 3.1 | lanthipeptide-class-i | 132,991 | 158,051 | N/A | N/A |
| Region 3.2 | NRP-metallophore,NRPS | 227,585 | 286,350 | mirubactin | 71% |
| Region 3.3 | terpene | 323,793 | 349,995 | hopene | 46% |
| Region 3.4 | NRPS,T1PKS | 518,191 | 576,596 | caerulomycin A | 8% |
| Region 3.5 | NRPS-like | 688,845 | 729,416 | youssoufenes | 14% |
| Region 3.6 | T3PKS | 753,301 | 794,356 | loseolamycins | 16% |
| Region 3.7 | NRP-metallophore,NRPS | 875,274 | 932,556 | polyoxypeptin | 32% |
| Region 5.1 | terpene | 148,627 | 169,832 | isorenieratene | 36% |
| Region 7.1 | nucleoside | 35,777 | 56,157 | tubercidin | 63% |
| Region 8.1 | other | 33,407 | 64,252 | polyoxypeptin | 16% |
| Region 31.1 | RRE-containing | 1 | 1,629 | N/A | N/A |
| Region 147.1 | RiPP-like | 1 | 1,133 | N/A | N/A |
| Region 193.1 | NRPS | 1 | 1,073 | xenematide | 100% |

**Table S5**. antiSMASH [3] output table of predicted biosynthetic gene clusters identified in *Streptomyces* sp. GSL17-113 (BioProject accession number PRJNA1066849 and BioSample number SAMN39507600).

| **Region** | **Type** | **From** | **To** | **Most similar known cluster** | **Similarity** |
| --- | --- | --- | --- | --- | --- |
| Region 2.1 | NRPS-independent-siderophore | 1 | 8,961 | desferrioxamine E | 100% |
| Region 2.2 | terpene | 168,897 | 205,590 | N/A | N/A |
| Region 2.3 | prodigiosin | 463,346 | 498,329 | tambjamine BE-18591 | 96% |
| Region 3.1 | NRPS-independent-siderophore | 54,094 | 69,396 | N/A | N/A |
| Region 3.2 | other,NRPS-like,T1PKS | 211,159 | 274,263 | sanglifehrin A | 18% |
| Region 5.1 | lanthipeptide-class-i | 15,694 | 40,339 | N/A | N/A |
| Region 5.2 | CDPS | 86,778 | 107,614 | a201a | 8% |
| Region 5.3 | other,nucleoside | 301,350 | 342,588 | pseudouridimycin | 68% |
| Region 5.4 | lanthipeptide-class-iii | 359,213 | 381,927 | SapB | 75% |
| Region 7.1 | lassopeptide | 169,240 | 192,222 | aborycin | 64% |
| Region 8.1 | terpene | 191,040 | 213,271 | geosmin | 100% |
| Region 12.1 | T2PKS | 1 | 38,642 | xantholipin | 55% |
| Region 12.2 | ectoine | 194,267 | 204,671 | ectoine | 100% |
| Region 13.1 | T1PKS | 1 | 35,210 | ibomycin | 29% |
| Region 15.1 | NRPS-like | 659 | 25,359 | meilingmycin | 6% |
| Region 15.2 | NRP-metallophore,NRPS | 38,409 | 86,998 | griseobactin | 53% |
| Region 17.1 | NRPS | 1 | 24,264 | dudomycin A | 47% |
| Region 18.1 | T1PKS,oligosaccharide | 1 | 72,435 | ibomycin | 58% |
| Region 21.1 | terpene | 1 | 24,664 | hopene | 61% |
| Region 23.1 | lanthipeptide-class-i, T1PKS,RiPP-like | 10,596 | 62,743 | 4-hexadecanoyl-3-hydroxy-2-(hydroxymethyl)-2H-furan-5-one | 54% |
| Region 25.1 | T1PKS | 4,760 | 55,801 | abyssomicins | 9% |
| Region 27.1 | T1PKS | 1 | 38,903 | reedsmycins | 20% |
| Region 29.1 | T1PKS | 4,666 | 27,498 | ulleungmycin | 5% |
| Region 31.1 | T1PKS | 1 | 23,355 | gargantulides | 26% |
| Region 38.1 | T1PKS | 1 | 3,734 | N/A | N/A |
| Region 42.1 | T1PKS | 1 | 2,991 | N/A | N/A |
| Region 43.1 | T1PKS | 1 | 2,320 | N/A | N/A |

**Table S6**. antiSMASH [3] output table of predicted biosynthetic gene clusters identified in *Streptomyces* sp. GSL17-111 (BioProject accession number PRJNA1077303 and BioSample number SAMN39507600).

| **Region** | **Type** | **From** | **To** | **Most similar known cluster** | **Similarity** |
| --- | --- | --- | --- | --- | --- |
| [Region 1.1](applewebdata://1E01C5BB-0304-43C8-980C-39FA3D5E130F#RANGE!r1c1) | NRPS-like,t erpene | 313,636 | 356,787 | [isorenieratene](https://mibig.secondarymetabolites.org/go/BGC0001456/1) | 87% |
| [Region 1.2](applewebdata://1E01C5BB-0304-43C8-980C-39FA3D5E130F#RANGE!r1c2) | [terpene](https://docs.antismash.secondarymetabolites.org/glossary/#terpene) | 426,548 | 446,579 | [hopene](https://mibig.secondarymetabolites.org/go/BGC0000663/1) | 30% |
| [Region 1.3](applewebdata://1E01C5BB-0304-43C8-980C-39FA3D5E130F#RANGE!r1c3) | [RiPP-like](https://docs.antismash.secondarymetabolites.org/glossary/#ripp-like) | 594,032 | 604,699 | [streptamidine](https://mibig.secondarymetabolites.org/go/BGC0002115/1) | 75% |
| [Region 1.4](applewebdata://1E01C5BB-0304-43C8-980C-39FA3D5E130F#RANGE!r1c4) | T1PKS, NRPS-like, NRPS | 664,449 | 792,443 | [piericidin A1](https://mibig.secondarymetabolites.org/go/BGC0001742/1) | 100% |
| [Region 1.5](applewebdata://1E01C5BB-0304-43C8-980C-39FA3D5E130F#RANGE!r1c5) | [NI-siderophore](https://docs.antismash.secondarymetabolites.org/glossary/#ni-siderophore) | 1,022,704 | 1,035,419 | N/A | N/A |
| [Region 1.6](applewebdata://1E01C5BB-0304-43C8-980C-39FA3D5E130F#RANGE!r1c6) | T1PKS, ladderane, arylpolyene | 1,153,760 | 1,251,115 | [o-dialkylbenzene 1/o-dialkylbenzene 2](https://mibig.secondarymetabolites.org/go/BGC0002441/1) | 61% |
| [Region 1.7](applewebdata://1E01C5BB-0304-43C8-980C-39FA3D5E130F#RANGE!r1c7) | T1PKS, PKS-like | 1,405,120 | 1,447,946 | [arsono-polyketide](https://mibig.secondarymetabolites.org/go/BGC0001283/1) | 56% |
| [Region 1.8](applewebdata://1E01C5BB-0304-43C8-980C-39FA3D5E130F#RANGE!r1c8) | [RiPP-like](https://docs.antismash.secondarymetabolites.org/glossary/#ripp-like) | 2,219,652 | 2,229,185 | N/A | N/A |
| [Region 1.9](applewebdata://1E01C5BB-0304-43C8-980C-39FA3D5E130F#RANGE!r1c9) | NRPS-like, lanthipeptide-class-i | 2,389,123 | 2,432,167 | [saquayamycin A](https://mibig.secondarymetabolites.org/go/BGC0001769/1) | 7% |
| [Region 1.10](applewebdata://1E01C5BB-0304-43C8-980C-39FA3D5E130F#RANGE!r1c10) | [NRPS](https://docs.antismash.secondarymetabolites.org/glossary/#nrps) | 2,473,340 | 2,517,374 | [diisonitrile antibiotic SF2768](https://mibig.secondarymetabolites.org/go/BGC0001574/1) | 66% |
| [Region 1.11](applewebdata://1E01C5BB-0304-43C8-980C-39FA3D5E130F#RANGE!r1c11) | NRPS,l anthipeptide-class-i, lanthipeptide-class-ii | 2,572,895 | 2,625,749 | [meilingmycin](https://mibig.secondarymetabolites.org/go/BGC0000093/1) | 3% |
| [Region 1.12](applewebdata://1E01C5BB-0304-43C8-980C-39FA3D5E130F#RANGE!r1c12) | [terpene](https://docs.antismash.secondarymetabolites.org/glossary/#terpene) | 3,053,834 | 3,076,053 | [geosmin](https://mibig.secondarymetabolites.org/go/BGC0001181/1) | 100% |
| [Region 1.13](applewebdata://1E01C5BB-0304-43C8-980C-39FA3D5E130F#RANGE!r1c13) | [T1PKS](https://docs.antismash.secondarymetabolites.org/glossary/#t1pks) | 3,437,737 | 3,523,150 | [piericidin A1](https://mibig.secondarymetabolites.org/go/BGC0001169/1) | 41% |
| [Region 1.14](applewebdata://1E01C5BB-0304-43C8-980C-39FA3D5E130F#RANGE!r1c14) | [other](https://docs.antismash.secondarymetabolites.org/glossary/#other) | 3,568,372 | 3,609,472 | [loseolamycin A1/loseolamycin A2](https://mibig.secondarymetabolites.org/go/BGC0002362/1) | 12% |
| [Region 1.15](applewebdata://1E01C5BB-0304-43C8-980C-39FA3D5E130F#RANGE!r1c15) | [CDPS](https://docs.antismash.secondarymetabolites.org/glossary/#cdps) | 3,765,499 | 3,786,239 | [primycin](https://mibig.secondarymetabolites.org/go/BGC0001447/1) | 5% |
| [Region 1.16](applewebdata://1E01C5BB-0304-43C8-980C-39FA3D5E130F#RANGE!r1c16) | [crocagin](https://docs.antismash.secondarymetabolites.org/glossary/#crocagin) | 4,498,082 | 4,520,894 | [massinidine](https://mibig.secondarymetabolites.org/go/BGC0002647/1) | 66% |
| [Region 1.17](applewebdata://1E01C5BB-0304-43C8-980C-39FA3D5E130F#RANGE!r1c17) | [lanthipeptide-class-iii](https://docs.antismash.secondarymetabolites.org/glossary/#lanthipeptide-class-iii) | 4,591,210 | 4,613,198 | [SapB](https://mibig.secondarymetabolites.org/go/BGC0000551/1) | 75% |
| [Region 1.18](applewebdata://1E01C5BB-0304-43C8-980C-39FA3D5E130F#RANGE!r1c18) | [NRPS-like](https://docs.antismash.secondarymetabolites.org/glossary/#nrps-like) | 4,731,050 | 4,773,444 | [indigoidine](https://mibig.secondarymetabolites.org/go/BGC0000727/1) | 27% |
| [Region 1.19](applewebdata://1E01C5BB-0304-43C8-980C-39FA3D5E130F#RANGE!r1c19) | [NI-siderophore](https://docs.antismash.secondarymetabolites.org/glossary/#ni-siderophore) | 5,033,830 | 5,050,078 | [nonactin/monactin/dinactin/trinactin/tetranactin](https://mibig.secondarymetabolites.org/go/BGC0000244/1) | 33% |
| [Region 1.20](applewebdata://1E01C5BB-0304-43C8-980C-39FA3D5E130F#RANGE!r1c20) | NRPS, T1PKS, ectoine | 5,123,462 | 5,187,600 | [ectoine](https://mibig.secondarymetabolites.org/go/BGC0002052/1) | 100% |
| [Region 1.21](applewebdata://1E01C5BB-0304-43C8-980C-39FA3D5E130F#RANGE!r1c21) | transAT-PKS, PKS-like | 5,203,524 | 5,285,218 | [lagriamide](https://mibig.secondarymetabolites.org/go/BGC0001646/1) | 9% |
| [Region 1.22](applewebdata://1E01C5BB-0304-43C8-980C-39FA3D5E130F#RANGE!r1c22) | lanthipeptide-class-iii, LAP,thiopeptide, T1PKS, NRPS | 5,710,143 | 5,998,596 | [stambomycin A/stambomycin B/stambomycin C/stambomycin D](https://mibig.secondarymetabolites.org/go/BGC0000151/1) | 68% |

**Table S7.** ^1^H-NMR resonances of tambjamine BE-18591, isolated from *Streptomyces* sp. GSL17-113. ^1^H chemical shifts are referenced to CDCL_3_.


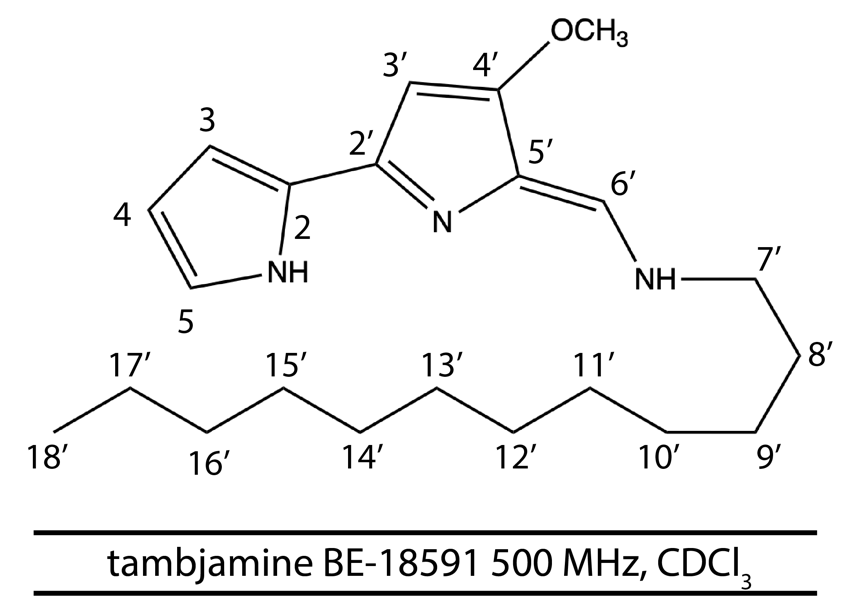


| Position | *δ* (^1^H), mult (*J* in Hz) |
| --- | --- |
| 2 | – |
| 3 | 6.74, m |
| 4 | 6.27, m |
| 5 | 7.09, s |
| 2’ | – |
| 3’ | 5.98, s |
| -OCH_3_ | 3.92, s |
| 4’ | – |
| 5’ | – |
| 6’ | 7.32, d (14.8) |
| 7’ | 3.45, t (6.54) |
| 8’ | 1.70, m |
| 9’ | 1.35, m |
| 10’ | 1.24, o.l.*^a^* |
| 11’ | 1.24, o.l. *^a^* |
| 12’ | 1.24, o.l. *^a^* |
| 13’ | 1.24, o.l. *^a^* |
| 14’ | 1.24, o.l. *^a^* |
| 15’ | 1.24, o.l. *^a^* |
| 16’ | 1.24, o.l. *^a^* |
| 17’ | 1.24, o.l. *^a^* |
| 18’ | 0.86, t (6.94) |

*^a^*o.l. = overlapping signal


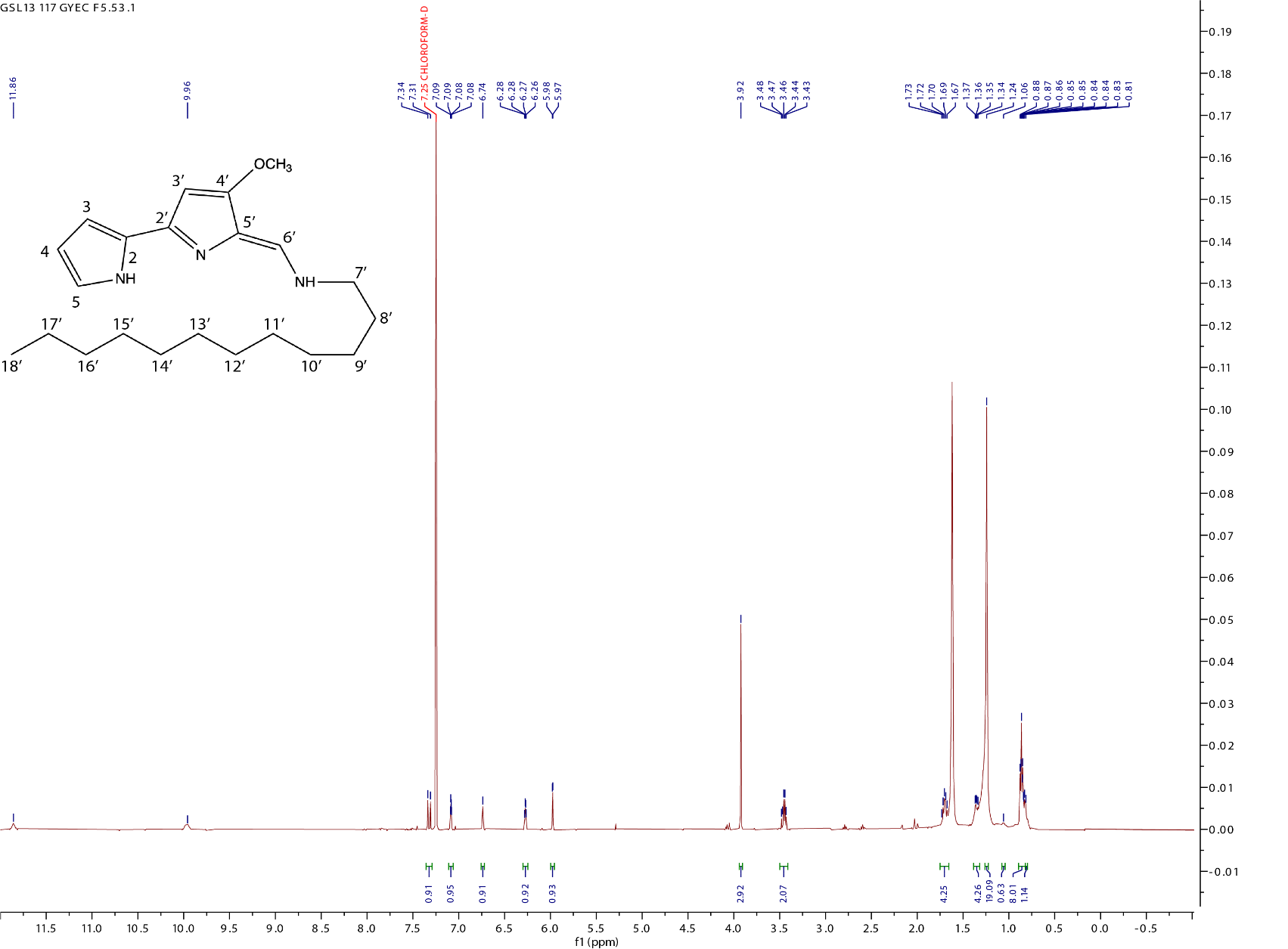


**Figure S7**. ^1^H NMR spectra of tambjamine BE-18591 in CDCl_3_ isolated from *Streptomyces* sp. GSL17-113.

**
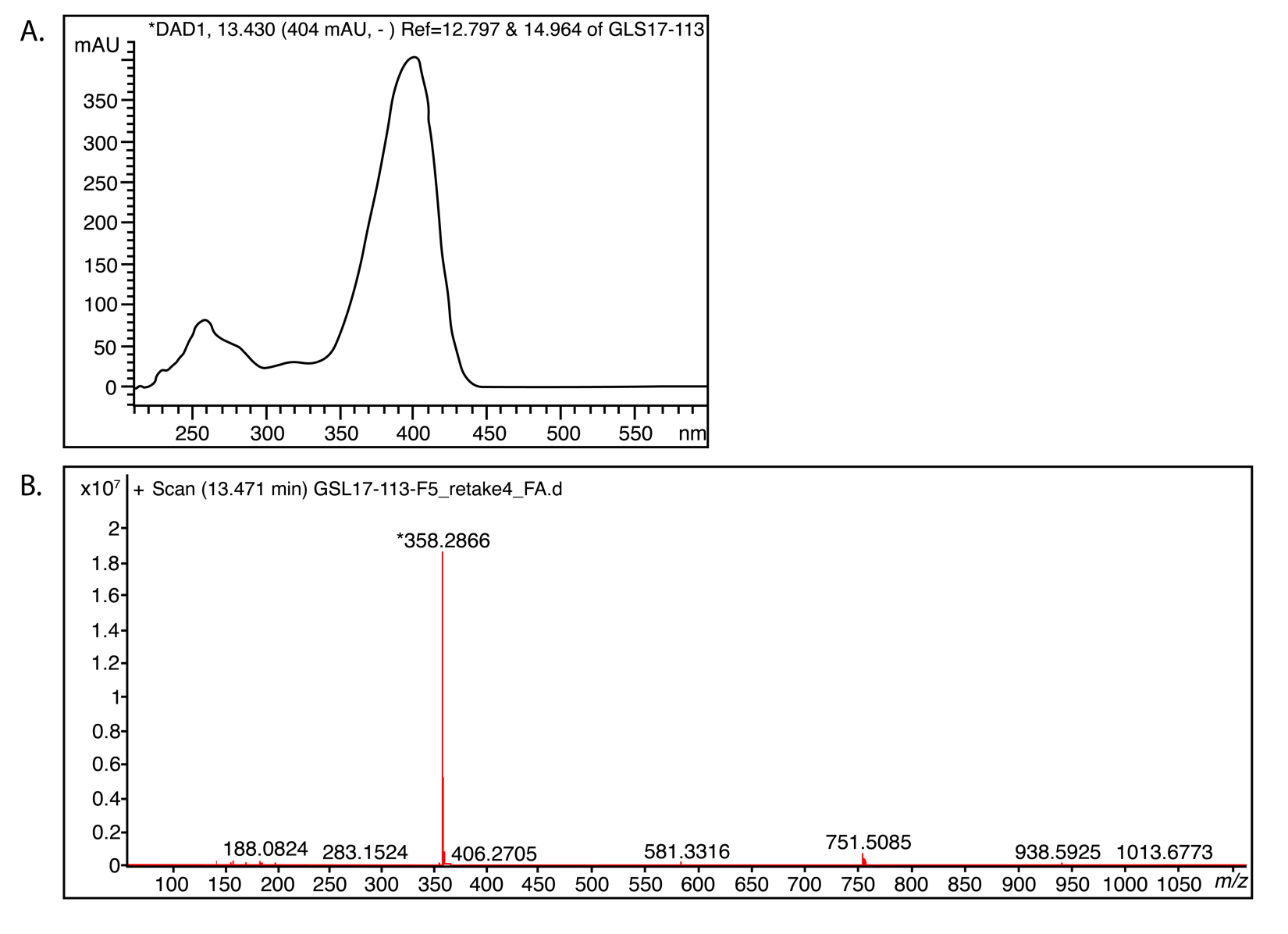
**

**Figure S8.** Identification of tambjamine BE-18591 from *Streptomyces* sp. GSL17-113. **A)** UV profile and **B)** HR-ESI-MS of tambjamine BE-18591. C_22_H_35_N_3_O [HRMS *m/z*: [M+H]^+^ calcd for C_22_H_36_N_3_O 358.2858; found 358.2866.

**Table S8**. Putative functions of the gene products of the tambjamine BE-18591 biosynthetic gene cluster in *Streptomyces* sp. GSL17-113 (GenBank Accession number PP179500).

| **Gene**  **Product** | **Total**  **Amino Acids** | **Proposed Function** | **Sequence Similarity**  **(Origin)** | **Similarity/**  **Identity (%)** | **Accession**  **Number** |
| --- | --- | --- | --- | --- | --- |
| TabD | 254 | AfsR/SARP family transcriptional regulator | *Streptomyces* | 99/99.6 | WP_016467654.1 |
| TabE | 611 | Aldehyde dehydrogenase | *Streptomyces albus* | 99/99.5 | TGG88512.1 |
| TabA | 444 | Aminotransferase class-III | *Streptomyces sp.* NRRL F-5639 | 95/99.5 | WP_031025583.1 |
| TabC | 162 | SRPBCC family protein | *Streptomyces* | 99/100 | WP_016467651.1 |
| TabP | 337 | β-ketoacyl-ACP synthase | *Streptomyces* | 99/100 | WP_016467650.1 |
| TabQ | 78 | Acyl-carrier protein | *Streptomyces* | 98/100 | WP_016467649.1 |
| TabR | 413 | β-ketoacyl-ACP synthase | *Streptomyces sp.* NRRL F-5639 | 99/99.5 | WP_031025584.1 |
| TabY | 105 | Hypothetical | *Streptomyces* | 99/100 | WP_016467647.1 |
| TabW | 380 | Acyl-CoA dehydrogenase | *Streptomyces* | 99/100 | WP_016467646.1 |
| TabX | 986 | Polyketide Synthase (KS-KS) | *Streptomyces albus* | 99/99.9 | WP_037611838.1 |
| TabO | 86 | Acyl-carrier protein | *Streptomyces* | 98/100 | WP_016467644.1 |
| TabN | 630 | Aminotransferase class I/II-fold pyridoxal phosphate-dependent enzyme | *Streptomyces* | 99/99.8 | WP_031174881.1 |
| TabM | 538 | D-alanine-poly(phosphoribitol) ligase | *Streptomyces* | 99/100 | WP_016467642.1 |
| TabJ | 280 | Alpha/beta fold hydrolase | *Streptomyces* | 99/99.6 | WP_016467641.1 |
| TabI | 355 | Class I SAM-dependent methyltransferase | *Streptomyces* | 99/100 | WP_016467640.1 |
| TabH | 900 | PEP/pyruvate-binding domain-containing protein | *Streptomyces* | 99/100 | WP_016467639.1 |
| TabU | 279 | 4'-phosphopantetheinyl transferase superfamily protein | *Streptomyces* | 86/100 | WP_041968511.1 |
| TabV | 420 | RedV protein | *Streptomyces* sp. NRRL F-5917 | 94/100 | WP_107047698.1 |

**Table S9.** Strains predicted to encode the tambjamine BE-18591 biosynthetic gene cluster identified in *Streptomyces* sp. GSL17-113 (BGC length: 25,345 bp).

| **Strain** | **Query Coverage (%)** | **Identity (%)** | **E-value** | **Accession** |
| --- | --- | --- | --- | --- |
| *Streptomyces albus* sp. HUT6047 | 100 | 99.70 | 0.0 | LC760459.1 |
| *Streptomyces. albus* sp. CAS922 | 100 | 99.55 | 0.0 | CP048875.1 |
| *Streptomyces. albus* sp. DSM 40763 | 100 | 99.77 | 0.0 | CP103060.1 |

**Supplemental References**

1. Love MI, Huber W, Anders S. Moderated estimation of fold change and dispersion for rna-seq data with deseq2. Genome Biol. 2014;15(12):550.

2. Alanjary M, Steinke K, Ziemert N. Automlst: An automated web server for generating multi-locus species trees highlighting natural product potential. Nucleic Acids Res. 2019;47(W1):W276-W82.

3. Blin K, Shaw S, Steinke K, Villebro R, Ziemert N, Lee SY, et al. Antismash 5.0: Updates to the secondary metabolite genome mining pipeline. Nucleic Acids Res. 2019;47(W1):W81-W7.
